# Octupolar Weyl superconductivity from electron–electron interaction

**DOI:** 10.1093/nsr/nwag326

**Published:** 2026-06-02

**Authors:** Zhiming Pan, Chen Lu, Fan Yang, Congjun Wu

**Affiliations:** Department of Physics, Xiamen University, Xiamen 361005, China; School of Physics, Hangzhou Normal University, Hangzhou 311121, China; School of Physics, Beijing Institute of Technology, Beijing 100081, China; New Cornerstone Science Laboratory, Department of Physics, School of Science, Westlake University, Hangzhou 310024, China; Institute for Theoretical Sciences, Westlake University, Hangzhou 310024, China; Key Laboratory for Quantum Materials of Zhejiang Province, School of Science, Westlake University, Hangzhou 310024, China; Institute of Natural Sciences, Westlake Institute for Advanced Study, Hangzhou 310024, China

**Keywords:** topological superconductivity, Weyl superconductivity, unconventional superconductor, strongly correlated systems

## Abstract

Weyl topological superconductivity (TSC) is an exotic superconducting state featuring topologically protected point gap nodes and Bogoliubov–Majorana Fermi arcs. However, its realization remains rare, often requiring complicated band structures or fine-tuned spin–orbit coupling. Here, we propose a universal and robust mechanism to generate Weyl TSC relying solely on 3D cubic lattice symmetry under repulsive electron–electron interaction. Standard group theory combined with Ginzburg–Landau analysis reveals that generalizing the $d_{x^2-y^2}$-wave pairing in the 2D square lattice, typically driven by repulsive interactions, to a 3 dimensional (3D) cubic lattice naturally yields a chiral $d_{3z^2-r^2}+ i d_{x^2-y^2}$ state. Unlike other time-reversal symmetry breaking pairings such as $p_x+ip_y$ (for example, $^3$He-A) and $d_{x^2-y^2}+id_{xy}$ under planar hexagonal symmetry, Cooper pairs with such a symmetry do not possess a net orbital angular momentum (OAM). Instead, they develop an octupolar $O_{xyz}$ component of OAM, which results in eight nodal points along the body diagonal directions, exhibiting an alternating distribution of monopole charges $\pm 1$. This naturally constitutes an octupolar Weyl TSC with non-trivial topology. Employing the single-orbital cubic Hubbard model as a prototype, we utilize a weak-coupling approach within the random-phase approximation, along with a strong-coupling analysis based on slave-boson mean-field theory and variational Monte Carlo simulations. Our numerical results consistently confirm the emergence of the $d+id$ Weyl TSC, highlighting the universality of this interaction-driven symmetry-protected mechanism. This finding simplifies the search for Weyl TSC, suggesting potential realization in cubic-lattice correlated superconductors and in cold-atom quantum simulation of the 3D Hubbard model upon suppression of its Néel antiferromagnetic phase.

## Teaser

This study reveals how electron repulsive forces in 3D cubic structures naturally generate Weyl superconductivity, driving new breakthroughs in unconventional superconductivity within 3D strongly correlated quantum systems.

## INTRODUCTION

The search for topological superconductivity (TSC) has attracted sustained interest, notably due to its potential to host Majorana fermions, which are crucial for fault-tolerant topological quantum computation [1–3]. Much of the focus has been on fully gapped TSCs [4–8], such as the *p*-wave Kitaev chain [9] and 2 dimensional (2D) chiral superconductors [10–16]. In 3 dimensional (3D) superconductors, there exists a particular type of TSC, the Weyl TSC [17–31], which is a superconducting analogue to the Weyl semimetal [32,33] exhibiting point gap nodes and surface Bogoliubov–Majorana Fermi arcs. The realization of Weyl TSC is exceptionally rare and presents formidable experimental challenges, primarily relying on the presence of complicated band structures and the precisely tuned, yet typically weak, relativistic single-particle effect of spin–orbit coupling (SOC) [34,35]. Here, we propose a universal and viable pathway to realize the Weyl TSC, which relies only on the cubic lattice symmetry under repulsive interactions.

Understanding unconventional superconductivity (SC) that emerges from many-body interactions is a central and persistent challenge in condensed matter physics. The emergence of such an interaction-driven superconducting state is fundamentally linked to the mechanism of high-$T_{\mathrm{c}}$ SC in the cuprates [36–41]. In the well-studied 2D square-lattice repulsive-interaction models such as the Hubbard or its descendant *t*−*J* model [42–46], antiferromagnetic (AFM) spin fluctuations usually drive $d_{x^2-y^2}$-wave pairing in which the signs of the order parameter along the *x*- and *y*-axes are opposite. Recently, quantum simulation of the Hubbard model [46–48] has been successfully extended to 3D optical lattices [49–51], leading to the observation of the AFM order [52] consistent with numerical simulations [53–58]. It is therefore crucial to explore the nature of the SC driven by AFM spin fluctuations upon suppressing the long-range AFM order [59–61].

While a direct extension of the sign structure of the order parameter from the 2D square-lattice $d_{x^2-y^2}$-wave pairing to a 3D cubic lattice inevitably encounters geometric frustration, a fundamental question arises: What is the appropriate extension of the 2D $d_{x^2-y^2}$-wave SC to 3D? Our analysis reveals that the answer lies in the chiral $d+id$ pairing state, formed by a 1:*i* superposition of $E_g$ components. This chiral $d+id$ pairing resolves the inherent frustration by establishing a $120^{\circ }$ phase difference among the *x*-, *y*-, and *z*-axes. It hosts topological point nodes, realizing a Weyl TSC. Furthermore, we demonstrate the robustness of this state by showing that it dominates in both the weak-coupling and strong-coupling regimes.

In this article, we systematically explore the nature of the pairing driven by repulsive interactions under 3D cubic symmetry. Pairing symmetry classification based on the irreducible representation (IRRP) of the $O_h$ point group, combined with a universal Ginzburg–Landau (G-L) analysis, indicates that the most natural repulsive-interaction-driven SC on a 3D cubic lattice is the $d_{3z^2-r^2}+id_{x^2-y^2}$ SC belonging to the $E_g$ IRRP. This 3D $d+id$ state exhibits point nodes and chirality, leading to the Weyl TSC. Taking the plain Hubbard model on the 3D cubic lattice as a prototype, we employ a weak-coupling analysis within the random-phase approximation (RPA), alongside a strong-coupling approach based on slave-boson mean-field theory (SBMFT) and variational Monte Carlo (VMC) simulations.

Our numerical data consistently show that the doubly-degenerate $E_g$ pairing is dominant at low and intermediate doping levels. Numerical calculations further show that the $d+id$ complex gap function with the octupolar $O_{xyz}$ component of orbital angular momentum (OAM) is energetically favored. The resulting quasiparticle spectrum exhibits eight point nodes in the bulk, with an alternating pattern of monopole charges $\pm 1$, realizing an octupolar Weyl TSC with topologically protected Fermi arcs. Our results apply to general systems with cubic symmetry under repulsive interactions. It opens the door to studying exotic quantum states in 3D driven by electron–electron interactions.

## RESULTS

### General symmetry-based analysis

**Figure 1.**
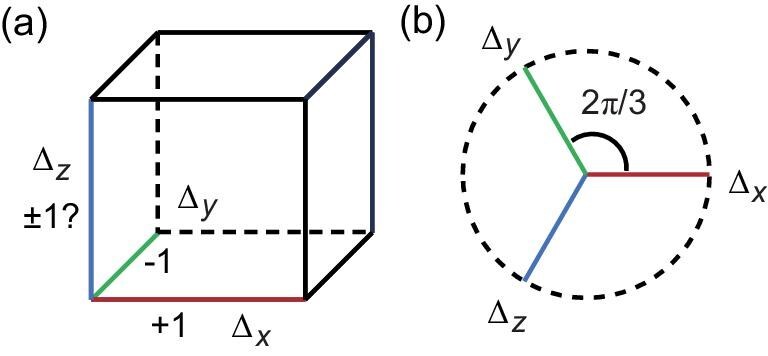
Cubic pairing gap function. (a) Gap functions $\Delta _x$, $\Delta _y$, and $\Delta _z$ along three axes in the 3D cubic lattice. (b) A phase difference of $120^{\circ }$ among the three pairing gaps represented in the complex plane. The pairing phases of 1, $e^{i2\pi /3}$, and $e^{i4\pi /3}$ correspond to the gaps $\Delta_x, \Delta_y, and \Delta_z$, respectively.

In 2D square lattices, AFM fluctuations usually drive the $d_{x^2-y^2}$-wave SC [42–44], whose pairing order parameter changes its sign between the *x* and *y* directions. However, directly extending this simple sign-alternating structure to a 3D cubic lattice immediately introduces a phase frustration. Specifically, it becomes impossible to simultaneously assign mutually opposite signs to the order parameter along all three directions, as illustrated in Fig. 1(a). Analogous to the frustrated magnetic order observed in triangular lattices, such frustration naturally leads to the possibility that the pairing phases along the three directions differ by $120^{\circ }$ from each other, as shown schematically in Fig. 1(b). This leads to the emergence of the $d+id$-wave SC, representing the natural generalization of the 2D $d_{x^2-y^2}$-wave SC to the 3D cubic lattice. In the following, we will theoretically and numerically verify this simple, yet powerful, symmetry argument.

The pairing symmetries on the cubic lattice are classified according to the IRRPs of the $O_h$ group [62–64] (see Section A of Supplemental Material (SM) for details). In particular, the *d*-wave channels on the 3D cubic lattice contain the 2-fold degenerate $E_g$ and the 3-fold degenerate $T_{2g}$ IRRPs. The $E_g$ IRRP, spanned by the doubly-degenerate $d_{3z^2-r^2}$ and $d_{x^2-y^2}$ basis functions, represents the 3D extension of the $B_{1g}$-IRRP corresponding to the $d_{x^2-y^2}$-wave pairing in 2D.

The degenerate pairings belonging to a given IRRP will be mixed below the critical temperature $T_{\mathrm{c}}$ to lower the G-L free-energy $\mathcal {F}$. Based on the above symmetry arguments, we consider the gap functions $\Delta _{1,2}$ representing the $d_{3z^2-r^2}$ and $d_{x^2-y^2}$ channels of the $E_g$ IRRP, respectively. Up to the quartic order, the only $U(1)\otimes O_h$ symmetry-allowed expression for $\mathcal {F}$ takes the following form [64,65]:


(1) \begin{eqnarray*}
\mathcal {F} &=& \alpha \big ( |\Delta _1|^2 +|\Delta _2|^2 \big ) +\beta _1 \big ( |\Delta _1|^2 +|\Delta _2|^2 \big )^2 \\
&& +\, \beta _2 \big | \Delta _1^{*} \Delta _2 - \Delta _2^{*} \Delta _1 \big |^2,
\end{eqnarray*}

where $\alpha < 0$ to stabilize the SC state, and $\beta _1> 0$ as required by thermodynamic stability. The relative phase between $\Delta _1$ and $\Delta _2$ is determined by the sign of $\beta _2$.

For $\beta _2< 0$, $\mathcal {F}$ is minimized when $\Delta _{1,2}$ exhibit equal magnitudes and a relative phase difference of $\pm \frac{\pi }{2}$, that is,


(2) \begin{eqnarray*}
\vec{\Delta }=(\Delta _1,\Delta _2) =\Delta (1,\pm i).
\end{eqnarray*}

This pairing symmetry is denoted as $d_{3z^2-r^2} \pm i d_{x^2-y^2}$ (hereafter abbreviated as $d+id$), exhibiting time-reversal symmetry breaking (TRSB). While the amplitude of this $d+id$ complex pairing remains invariant under all operations of the cubic point group $O_h$, its phase transforms non-trivially under the $C_3$ rotation of the cubic lattice. Specifically, a $120^{\circ }$ rotation about the body diagonal (for example, $x\rightarrow y\rightarrow z$) induces a $240^{\circ }$ shift in the superconducting phase. This leads to a $120^{\circ }$ phase difference between the pairings along the three directions ($x,y,z$), a feature consistent with Fig. 1(b). This $C_3$ phase transformation determines that the superconducting order parameter must vanish along the four equivalent body diagonal axes, thereby creating a total of 8 gap nodes (specifically, two point nodes along each axis) on the Fermi surface [23,63].

The 3D complex $d+id$ state is inherently topological, a feature evidenced by the non-trivial phase winding around the body diagonal. This characteristic is analogous to the well-established phase winding around the *z*-axis perpendicular to the plane in the 2D chiral $d_{x^2-y^2}+i d_{xy}$ TSC on the triangular or hexagonal lattice [11–16,64], implying its topological nature. Unlike the fully gapped 2D $d_{x^2-y^2}+i d_{xy}$ state, the 3D $d+id$ state obtained here generally hosts point nodes, as analyzed below. The condition for gap closing of the Bogoliubov spectrum, $E(\mathbf {k})=\sqrt{\varepsilon ^2(\mathbf {k})+|\Delta (\mathbf {k})|^2}$, requires the simultaneous vanishing of the bare electronic spectrum $\varepsilon (\mathbf {k})=0$ (defining the Fermi surface) and the complex gap function $\Delta (\mathbf {k})=0$. The latter imposes two constraints, stemming from the real and imaginary parts of the complex order parameter. In 2D momentum space, with only two free parameters $(k_x,k_y)$, satisfying these three independent conditions simultaneously is generally impossible, hence the system remains fully gapped [11–13,64]. In sharp contrast, the 3D complex $d+id$ state offers three momentum degrees of freedom, $(k_x,k_y,k_z)$. This naturally allows the three conditions to be satisfied at isolated points in momentum space, leading to the formation of point nodes in the Bogoliubov spectrum. The presence of point nodes combined with the non-trivial topology leads to the Weyl TSC.

Conversely, if the coupling parameter $\beta _2> 0$, the free-energy $\mathcal {F}$ favors a state where $(\Delta _1,\Delta _2)$ share the same phase, and their magnitudes are determined by higher-order terms in the free energy, leading to a purely (1, 0) or (0, 1) configuration. This is an example of nematic pairing symmetry, hereafter denoted as the $d+d$ state, which spontaneously breaks the system’s full $O_h$ symmetry. Crucially, the $d+d$ state breaks the $C_3$ rotational symmetry about the body diagonal, providing a unique experimental signature that distinguishes it from the $d+id$ state. For this real nematic $d+d$ state, the gap of the Bogoliubov spectrum closes when both the bare electronic spectrum $\varepsilon (\mathbf {k})$ and the gap function $\Delta (\mathbf {k})$ vanish simultaneously. Thus, in the 3D nematic state, the three momentum degrees of freedom allow these two constraints to be satisfied along continuous curves on the Fermi surface, resulting in the presence of line nodes.

The conditions and physical properties of the $d+id$ and $d+d$ pairing states are summarized and compared in Table 1. The distinct nodal structures of these two states give rise to different low-energy Bogoliubov quasiparticle density of states (DOS) behaviors. The 8 point nodes of the 3D $d+id$ state lead to a quadratic energy dependence, $N(E)\propto E^2$. In contrast, the line nodes of the nematic $d+d$ state result in a linear dependence, $N(E)\propto E$. These distinct quasiparticle excitation spectra provide direct and observable signatures that can be readily distinguished in future thermodynamic and spectroscopic measurements, such as specific heat and Knight shift, etc. See more details of the G-L analysis in Section A of SM.

**Table 1.** Characteristic properties of the chiral $d+id$ and nematic *d*-states belonging to the $E_g$ representation on a 3D cubic lattice.

Pairing	$d_{3z^2-r^2}\pm id_{x^2-y^2}$	$d_{3z^2-r^2}$ or $d_{x^2-y^2}$
Condition	$\beta _2 < 0$	$\beta _2 > 0$
TRS	Broken	Preserved
Residual symmetry	$C_3$ under gauge equivalence	orthorhombic (nematic)
Nodal structure	8 points	line-like
$N(E)$	$\propto E^2$	$\propto |E|$

The Ginzburg–Landau parameter $\beta _2$ governs the ground state. The $C_3$ rotation is defined around any of the body diagonal lines. $N(E)$ represents the low-energy quasiparticle density of states.

While evaluating the G-L parameter $\beta _2$ microscopically is generally complex, weak-coupling theory typically favors the $d+id$ pairing. The magnitude of the gap function for this state preserves the full cubic symmetry of $O_h$, exhibiting nodal points on the Fermi surface. In contrast, the $d+d$ pairing exhibits an anisotropic gap function with nodal lines. Hence, the amplitude distribution of the $d+id$ state is more uniform. Since the mean-field free energy is a convex function of the gap amplitude, it naturally favors a uniform distribution over the Fermi surface [17,66]. Therefore, the $d+id$ pairing state is energetically preferred in the weak-coupling regime, ultimately giving rise to the Weyl TSC. To verify this symmetry-based conclusion and provide concrete numerical evidence, we now investigate a prototypical lattice model.

### A prototypical model and numerical results

The simplest single-orbital repulsive Hubbard model is adopted as a prototype for illustrating the pairing problem on the 3D cubic lattice:


(3) \begin{eqnarray*}
H = -t \sum _{\langle i,j\rangle \sigma } c_{i\sigma }^{\dagger } c_{j\sigma } -\mu \sum _i n_i +U \sum _i n_{i\uparrow } n_{i\downarrow },
\end{eqnarray*}

where $c_{i\sigma }^{\dagger }$ ($c_{i\sigma }$) is the creation (annihilation) operator for a fermion with spin $\sigma$ at site *i*, respectively; $n_{i\sigma }=c_{i\sigma }^{\dagger }c_{i\sigma }$ represents the onsite fermion number operator for spin $\sigma$ and $n_i$ is the total fermion number operator; *t* is the hopping integral; *U* is the on-site Hubbard repulsion; $\langle i,j\rangle$ represents the nearest-neighbor (NN) bonds; the chemical potential *μ* controls the fermion filling and the system is particle–hole symmetric at $\mu =U/2$. We focus on the hole doping regime, where the hole doping level $\delta$ is defined as the deviation from half-filling: $\delta =1-n\ge 0$. Here, *n* is the average electron filling per site, with $n=1$ corresponding to half-filling.

At half-filling, the 3D Hubbard model exhibits long-range Néel AFM order [53–58] in both limits of $U/t\rightarrow 0$ (weak coupling) and $U/t \rightarrow +\infty$ (strong coupling). In the weak-coupling regime, the Fermi surface exhibits perfect nesting at the wavevector $\mathbf {Q}=(\pi ,\pi ,\pi )$, leading to the AFM order. In the strong-coupling regime, the system is Mott-insulating. The superexchange interaction is responsible for the low-energy physics as described by the AFM Heisenberg model, which shows long-range AFM ordering at low temperatures [40,67].

Upon doping, the AFM order is gradually suppressed and completely disappears above a critical doping level. However, the short-range AFM fluctuations persist, providing the pairing glue for SC. In the following, we investigate the pairing instabilities in the weak-coupling regime utilizing the RPA method and in the strong-coupling regime using the SBMFT and VMC approaches.

In the weak-coupling regime, the effective attraction between a pair of fermions with opposite momenta is mediated by the exchange of spin fluctuations, a mechanism captured in the RPA approach [15,16,41,59,68–74]. We employ the RPA method to calculate the pairing eigenvalues ($\lambda$), comparing them across various symmetry channels to identify the dominant pairing instability, which corresponds to the channel with the largest eigenvalue (see Section B of SM for details). Since the AFM ordering develops near half filling, we avoid the extremely low doping regime in which SC is suppressed, and focus on the low and intermediate hole doping regime $\delta \in (0.05, 0.5)$.

**Figure 2.**
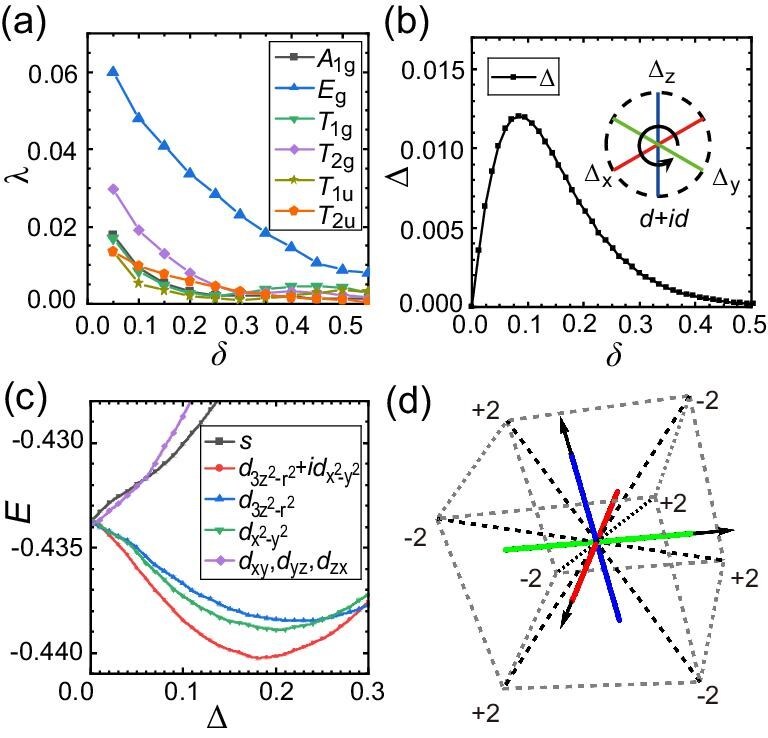
Numerical results from both weak- and strong-coupling approaches consistently show the dominance of the $E_g$ symmetry pairing. (a) Random-phase approximation result of the pairing eigenvalues $\lambda$ vs. $\delta$ for various pairing symmetries, calculated with $U/t=1.8$. (b) Slave-boson mean-field theory result of the pairing gap $\Delta$ vs. $\delta$ for the *t*−*J* model, using $J/t=0.4$ ($U/t=10$). (c) Variational Monte Carlo results showing the energy for different pairing symmetries, using $J/t=0.25$ and $\delta =0.1$. The results are obtained from minimizing the energy with respect to *μ* for a fixed $\Delta$. (d) The vorticity projection along the body diagonal lines displays an alternating pattern of $\pm 2$. A Cooper pair with such a symmetry carries a non-zero OAM octupolar component $O_{xyz}$. The corresponding gap function features 8 nodes on the Fermi surface.

The numerical results of pairing eigenvalues in various symmetry channels are presented in Fig. 2(a) as a function of $\delta$ for $U/t=1.8$. It clearly shows that the $E_g$ IRRP dominates in the low doping regime, that is, the doubly-degenerate singlet pairing symmetry of the $(d_{3z^2-r^2},d_{x^2-y^2})$ character. This pairing symmetry is favored by the strong AFM fluctuations in this regime, which arise from the nearly nested Fermi surface [59–61].

We have further performed detailed mean-field calculations subsequent to the RPA study, including both complex $d+id$ and real $d+d$ pairing states within the $E_g$ symmetry. These calculations show that the complex mixed pairing state is energetically favorable, which implies that $\beta _2< 0$, consistent with the weak-coupling analysis that favors the $d+id$ pairing. (For details and numerical data, see Section B of SM).

In the strong-coupling regime, the superexchange interaction drives the local pairing of the resonant-valence-bond type, which upon doping develops long-range phase coherence, leading into SC [37,40]. This physical picture can be accurately captured within theoretical treatments of the effective *t*−*J* model, employing approaches such as the SBMFT [40,42] or VMC simulation [75,76]. Indeed, the low-energy physics in this strong-coupling limit is described by the effective *t*−*J* model:


(4) \begin{eqnarray*}
H_{tJ}&=&-t \sum _{\langle i,j\rangle \sigma } c_{i\sigma }^{\dagger } c_{j\sigma } -\mu \sum _i n_i \\
&&+\,J\sum _{\langle i,j\rangle } \Big (\mathbf {S}_i\cdot \mathbf {S}_j -\frac{1}{4} n_i n_j \Big ),
\end{eqnarray*}

where $\mathbf {S}_i$ is the spin operator at site *i* and $J=4t^2/U$. Note that the no-double-occupancy constraint is imposed on the Hilbert space, which can be maintained in the SBMFT (see Section C of SM for details).

The AFM superexchange interaction *J* induces the NN pairings $\Delta _{x,y,z}$ along the $x,y$, and *z*-directions, respectively. Calculations based on SBMFT reveal that the NN pairings take the form of $(\Delta _x,\Delta _y,\Delta _z)=\Delta (1, e^{i2\pi /3},e^{ i4\pi /3})$ or its symmetry equivalences. This corresponds to the $d+id$ symmetry consistent with the RPA results in the weak-coupling regime. The doping $\delta$-dependence of the gap amplitude $\Delta$ is depicted in Fig. 2(b), which shows that as the doping increases, the superconducting order quickly emerges and then gradually decreases. Further SBMFT simulated results are provided in Section C of SM.

To corroborate the SBMFT results, VMC simulations have been also performed on the *t*−*J* model (see Section D of SM for details). The trial wavefunction for a SC state is constructed as a Gutzwiller-projected BCS function $|G\rangle ={-8}\hat{P}_G|\mathrm{BCS}\rangle$, where the Gutzwiller projector $\hat{P}_G$ strictly enforces the no-double-occupancy constraint. This results in a physical Hilbert space limited to empty, spin-$\uparrow$ or spin-$\downarrow$ occupancy at each site, appropriate for the hole-doped regime. Then, for each candidate pairing symmetry, the ground state energy $E=\langle {G}|H_{tJ}|{G}\rangle /\langle {G}|{G}\rangle$ evaluated for a given pairing gap function $\Delta (\mathbf {k})$, is optimized with respect to the chemical potential *μ*.

The pairing gap function $\Delta (\mathbf {k})=\Delta f(\mathbf {k})$ incorporates form factors $f(\mathbf {k})$ that correspond to specific pairing symmetries, consistent with the IRRPs of $O_h$ group. The AFM superexchange *J*-term acts only between NNs, naturally favoring singlet and short-range pairings. We focus on the NN and next-nearest-neighbor (NNN) range pairing configurations (see Section D of SM), encompassing various *s*- and *d*-wave states.

Specifically, for the $E_g$ IRRP, the G-L analysis suggests either the $d+d$ nematic pairing (with a 1:0 or 0:1 superposition) or the complex $d+id$ state (with 1:*i* mixing). Figure 2(c) presents a summary of the VMC energy comparison across various dominant pairing symmetries. Other possibilities were also tested but are not depicted, as they yield energies higher than that of the normal state. These results consistently demonstrate that the $d+id$ pairing state is energetically the most favorable, thereby providing strong support for its dominance in the strong-coupling regime.

### Octupolar and Weyl TSC

The $d+id$ pairing realized with the $E_g$-orbital basis differs fundamentally from the $p_x+ip_y$-type of $^3$He-A [77,78], as well as the $d_{x^2-y^2}+i d_{xy}$-type in triangular or hexagonal lattices [11–16,64]. The pairing symmetries of the latter two cases carry definite OAM quantum number of $L_z=1$ and 2, respectively. In contrast, the $E_g$ bases ($d_{3z^2-r^2}$ and $d_{x^2-y^2}$) characterizing the relative wave function of a Cooper pair can be expressed in terms of the *d*-wave ($l=2$) OAM representations as


(5) \begin{eqnarray*}
&& |d_{3z^2-r^2}\rangle =|2,0\rangle , \\
&&|d_{x^2-y^2}\rangle =\frac{1}{\sqrt{2}} \big (|2,2\rangle +|2,-2\rangle \big ).
\end{eqnarray*}

Here, $|l,m\rangle$ denotes the spherical harmonic basis $Y_{l,m}({-8}\hat{\mathbf {k}})$ that describes the relative motion of the Cooper pair in momentum space. Notably, these two basis states cannot be interconverted via simple angular momentum raising/lowering operators $\hat{L}_\pm ={-8}\hat{L}_x\pm i{-8}\hat{L}_y$, since these operators only shift the azimuthal quantum number *m* by 1. The resulting chiral state, $|d+id\rangle =\frac{1}{\sqrt{2}}(|d_{3z^2-r^2}\rangle +i|d_{x^2-y^2}\rangle )$, carries no net OAM, nor does it possess any traceless quadrupole tensor components; mathematically, their expectation values vanish, that is, $L_{\mu }\equiv \langle d+id|{-8}\hat{L}_{\mu }|d+id\rangle$ and $Q_{\mu \nu }\equiv \langle d+id|\frac{1}{2}({-8}\hat{L}_{\mu } \hat{L}_{\nu }+{-8}\hat{L}_{\nu } \hat{L}_{\mu })-\frac{1}{3}{-8}\hat{\mathbf {L}}^2\delta _{\mu \nu }|d+id\rangle$ ($\mu ,\nu =x,y,z$).

The absence of conventional OAM and quadrupole moment points to a hidden order. Specifically, when viewed along the body diagonal directions, the system exhibits an alternating vorticity of $\pm 2$ under $C_3$ rotations. This signifies an octupolar pattern, as illustrated in Fig. 2(d). Indeed, a Cooper pair with such a symmetry carries a finite OAM octupole component. Defining the OAM octupole operator as $\hat{O}_{xyz}= \frac{1}{3!} \sum _{P} \hat{L}_{\mu } \hat{L}_{\nu } \hat{L}_{\rho }$ (with *P* representing the permutations of $x,y,z$), the expectation value is non-zero: $O_{xyz}\equiv \langle d+id|{-8}\hat{O}_{xyz}|d+id\rangle =-\sqrt{3}$.

The octupolar $d+id$-pairing obtained in the low and intermediate doping regimes exhibits interesting symmetry and topological properties. Without loss of generality, its gap function in momentum space is expressed as


(6) \begin{eqnarray*}
\Delta (\mathbf {k}) = \Delta \big ( \cos k_x +e^{i\frac{2\pi }{3}} \cos k_y +e^{i\frac{4\pi }{3}} \cos k_z\big ).
\end{eqnarray*}

Its nodal directions appear along the body diagonals $(\pm 1, \pm 1, \pm 1)$, consistent with the octupolar symmetry. In comparison, the $p_x+ip_y$ pairing can be viewed as the dipolar symmetry exhibiting nonzero $L_z$ and its nodes are located along the *z*-axis. In the Nambu representation, the Bogoliubov-de Gennes Hamiltonian is given by


(7) \begin{eqnarray*}
H_{\mathrm{BdG}} &=& \sum _{\mathbf {k}} {\left(c_{\mathbf {k}\uparrow }^{\dagger }\quad c_{-\mathbf {\downarrow }} \right)} H(\mathbf {k})
\left(\begin{array}{c}
{c_{\mathbf{k}\uparrow}}\\
c^{\dagger}_{-\mathbf {k}\downarrow }
\end{array}\right),\\
H(\mathbf {k}) &=&\varepsilon (\mathbf {k}) \tau _3 +\mathrm{Re}\Delta (\mathbf {k}) \tau _1 -\mathrm{Im}\Delta (\mathbf {k}) \tau _2, \\
\end{eqnarray*}

where $\varepsilon (\mathbf {k})=-2t(\cos k_x+\cos k_y +\cos k_z)-\mu$ is the kinetic energy and $\tau _{i}$ are the Pauli matrices in Nambu space. The quasiparticle dispersion exhibits 8 nodal points located at the intersections of the body diagonal directions and the Fermi surface, that is, $\mathbf {k}_{\mathrm{node}}=\frac{k_{\mathrm{F}}}{\sqrt{3}}(\pm 1,\pm 1,\pm 1)$, where $k_{\mathrm{F}}$ is the magnitude of the Fermi wavevector along the body diagonal directions. Due to TRSB, these nodal points are identified as ‘Weyl nodes’ for the Bogoliubov quasiparticles [17,33].

Near the nodal points, the quasiparticles exhibit the following low-energy linear dispersion relation:


(8) \begin{eqnarray*}
H(\mathbf {p})= (\mathbf {v}_1\cdot \mathbf {p})\tau _1 +(\mathbf {v}_2\cdot \mathbf {p})\tau _2 +(\mathbf {v}_3\cdot \mathbf {p})\tau _3,
\end{eqnarray*}

where $\mathbf {p}\equiv \mathbf {k}-\mathbf {k}_{\mathrm{node}}$ is the relative momentum with respect to $\mathbf {k}_{\mathrm{node}}$ and $\mathbf {v}_i$ ($i=1,2,3$) are velocity vectors characterizing the local geometry of the Bogoliubov–Weyl cone (see Section E of SM for details).

**Figure 3.**
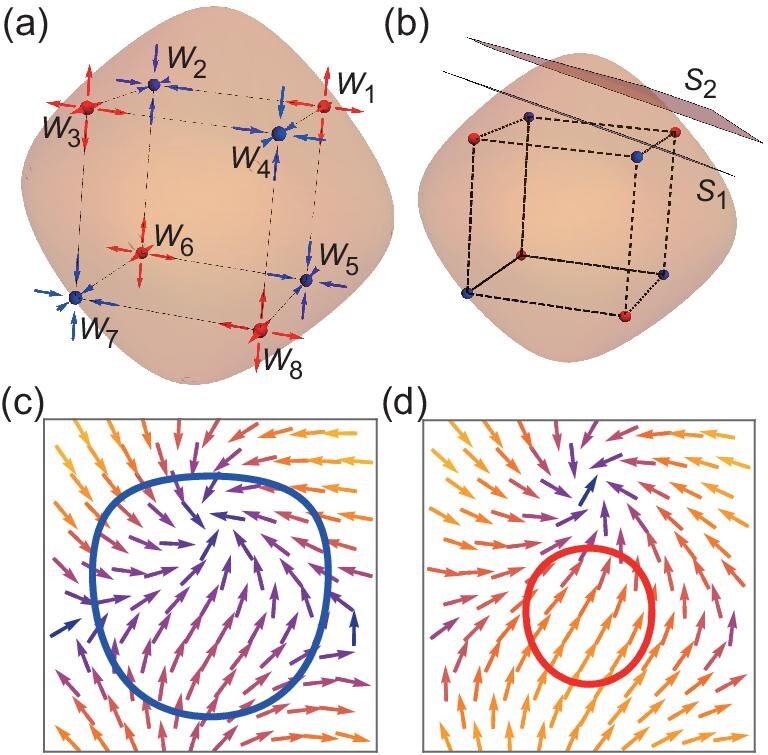
Momentum space topology of octupolar Weyl superconductor. (a) The 8 Weyl points around the Fermi surface (represented by the shadow region) with alternating monopole charges exhibit the octupolar pattern. The circles indicate Weyl points acting as positive or negative magnetic monopoles. (b) Cross-sections ($S_1$ and $S_2$) with topologically non-trivial and trivial 2D pairing configurations, shown in (c) with $C=1$ for $S_1$ and (d) with $C=0$ for $S_2$, respectively. The closed curves in (c) and (d) represent the two intersections, $S_1$ and $S_2$, of the 3D Fermi surface with the two planes in (b), respectively. Small arrows indicate the orientations of the pairing phase.

The 3D chirality (monopole charge) associated with a given Weyl node can be directly calculated as $C=\mathrm{Sgn}\big [\mathbf {v}_1\cdot \big ( \mathbf {v}_2\times \mathbf {v}_{3} \big ) \big ]=\pm 1$ [23,32]. The Weyl node at $\mathbf {k}_{\mathrm{node}}$ with $C=+1$  $(-1)$ acts as a positive (negative) magnetic monopole in momentum space, and its chirality is determined by the octupolar vorticity along $\mathbf {k}_{\mathrm{node}}$. Consequently, the monopole charges of the 8 Weyl nodes exhibit an octupolar pattern, as illustrated in Fig. 3(a). This low-energy effective theory suggests a Weyl superconductor [17], which is a superconducting counterpart to the Weyl semimetal.

The emergence of Bogoliubov–Weyl points in this $d+id$ pairing state is topologically protected. Consider an arbitrary 2D cross-section that intersects the 3D Fermi surface while avoiding the Weyl points. It results in a 2D gapped superconductor, which can be topologically either trivial or non-trivial depending on the location of the cross-section. This topology is characterized by a 2D Chern number, which describes the pairing phase winding around the 2D Fermi surface [4,5]. As illustrated in Fig. 3(b), shifting the section plane across a Weyl point alters this invariant from $C=1$ before crossing the point (Fig. 3(c)) to $C=0$ afterward (Fig. 3(d)). In other words, the 2D SC within the slice transitions from a topological phase to a trivial one. Hence, an intermediate Weyl node is unavoidable.

According to the bulk-boundary correspondence, the Weyl SC exhibits non-trivial Fermi-arc surface states, which are the Majorana arcs of the Bogoliubov quasiparticles. Similar to Weyl semimetals, the configuration of these surface Fermi arcs depends sensitively on the surface orientation. In particular, on the (100) surface, the projections of Weyl nodes with opposite monopole charges overlap and cancel each other, resulting in the absence of protected Fermi arcs.

**Figure 4.**
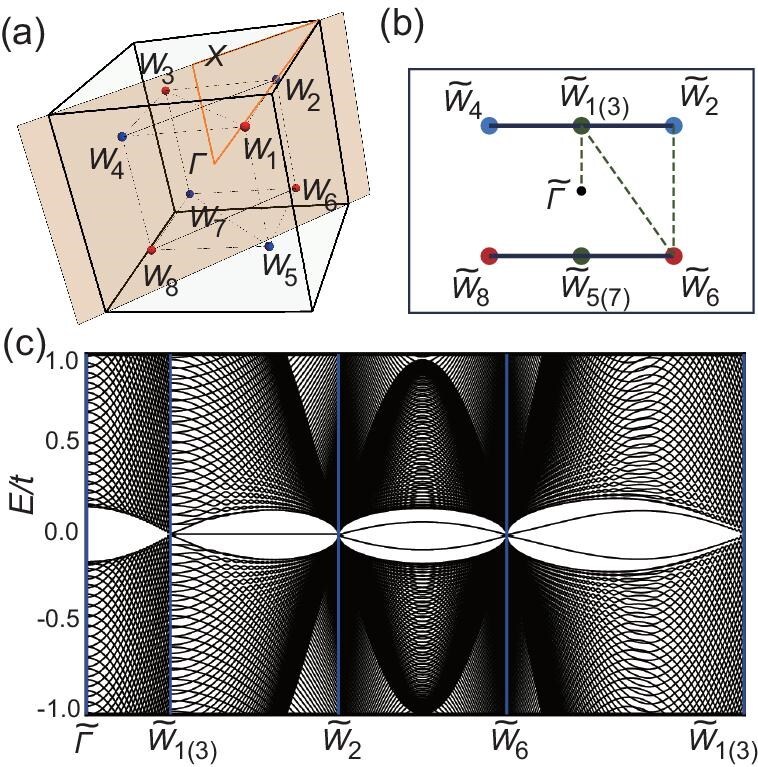
Weyl modes and surface Fermi arc. (a) Schematic diagram for the (110)-surface (shadow plane). (b) Projected Weyl nodes $\tilde{W}_i$ in the (110) surface Brillouin Zone and the Fermi arcs (solid lines), where $\tilde{W}_{1(3)}$ and $\tilde{W}_{5(7)}$ each represent the overlap of two bulk nodes due to the projection geometry. (c) Energy spectrum along the path $\tilde{\Gamma }-\tilde{W}_{1(3)}-\tilde{W}_2-\tilde{W}_6-\tilde{W}_{1(3)}$ marked in (b) (dashed lines) for $|\Delta |=0.1t$ and $\mu =-3t$ based on Equation (7).

Specifically, we consider the (110) surface shown in Fig. 4(a). The projection of the 8 Weyl nodes $W_i (i=1,\cdots , 8)$ onto this surface results in 6 distinct points $\tilde{W}_i$ marked in the surface Brillouin Zone (BZ) shown in Fig. 4(b), due to the overlap of two pairs of nodes. To explicitly reveal the gapless surface modes originating from the projected Weyl points, we calculate the surface spectrum along the path $\tilde{\Gamma }-{-6}\tilde{W}_{1(3)}-{-6}\tilde{W}_2-{-6}\tilde{W}_6-{-6}\tilde{W}_{1(3)}$ in the surface BZ (the dashed lines in Fig. 4(b)) as shown in Fig. 4(c). Particularly, along the path from $\tilde{W}_{1(3)}$ to $\tilde{W}_2$, there exist a continuous band of gapless modes forming the Fermi arc $\tilde{W}_{1(3)}-{-6}\tilde{W}_2$ exhibited in Fig. 4(b). The other Fermi arcs in Fig. 4(b) are obtained by symmetry. Note that the Fermi arcs $\tilde{W}_{1(3)}-{-6}\tilde{W}_2$ and $\tilde{W}_{1(3)}-{-6}\tilde{W}_4$ are connected at the $\tilde{W}_{1(3)}$ point on the (110) surface.

Crucially, these Majorana Fermi arcs provide a definitive signature to distinguish the $d+id$ Weyl TSC from the topologically trivial $d+d$ nematic state. While both states feature bulk nodal excitations, only the $d+id$ phase supports protected gapless surface modes. These surface states enhance the low-energy local DOS at the boundary. By mapping this local DOS and the associated quasiparticle interference (QPI) patterns, scanning tunneling microscopy and spectroscopy (STM/STS) can serve as an experimental probe for the underlying pairing symmetry.

## CONCLUSION AND DISCUSSION

Our comprehensive investigation focuses on the SC state arising solely from repulsive electron-electron interactions in a system with 3D cubic symmetry. Crucially, symmetry considerations combined with a G-L analysis strongly favor a TRSB $d+id$ SC, which belongs to the $E_g$  *d*-wave IRRP. Consistent with this, numerical results from both weak-coupling and strong-coupling calculations, using the Hubbard model as a prototype, unambiguously reveal the emergence of this TRSB $d+id$ pairing upon carrier doping. This robust state signifies a novel octupolar Weyl TSC, characterized by topologically protected Bogoliubov–Weyl nodes and surface Majorana–Fermi arcs. Our findings obviate the need for complex mechanisms such as SOC. They demonstrate that simple correlated cubic systems provide a direct route to realizing correlation-driven Weyl TSCs. This opens a new avenue for topological material design in a broad class of compounds with cubic symmetry.

The RPA calculations in the weak-coupling regime also indicate the emergence of the $T_{2g}$ singlet pairing symmetry ($d_{xy},d_{xz},d_{yz})$, or the triplet $T_{1u}$ symmetry (the *p*-wave) at sufficiently high doping levels $\delta > 0.6$. Crucially, the resulting pairing ground states are also expected to exhibit TRSB [79] with exotic properties. Similar to the complex $d+id$ state, these TRSB superconductors are generally predicted to host topological point nodes. They can also realize the Weyl TSCs, offering alternative candidate states beyond the $E_g$ channel. These exotic ground states may also feature other non-trivial phenomena, such as the sextetting order (charge-$6e$) [80–84]. Detailed results supporting these findings are provided in the Section B of SM.

The theoretical proposal for the interaction-driven Weyl TSC immediately points to cubic materials as ideal experimental platforms. Previous studies on cubic compounds, such as UBe$_{13}$ [85], CeIn$_3$ [86], and CsW$_2$O$_6$ [87] have suggested the existence of unconventional SC [88–91]. Most notably, the cubic spinel LiTi$_2$O$_4$ is a compelling candidate, which exhibits experimental signatures of *d*-wave pairing SC belonging to the $E_g$ IRRP [92]. This material exhibits strong electron-correlation properties driven by the Ti $3d$-orbital electrons [93–95], a characteristic that aligns with the physics underlying our proposal. It’s also worth noting that the resonating-valence-bond scenario has been previously suggested as a potential SC mechanism for this compound [37,96]. Recent experiments indicate that the pairing amplitude in this material maintains the full $O_h$ rotational symmetry [92]. This observation provides compelling evidence supporting the proposed $d+id$ Weyl TSC.

Moreover, we predict further hallmark features, such as the 8 bulk point gap nodes along the body diagonal directions and topologically protected surface Fermi arcs, which are prime targets for experimental verification via angle-resolved photo-emission spectroscopy and STM. This work re-establishes the role of cubic superconductors, demonstrating their profound potential to realize the exotic Weyl TSC and calling for dedicated experimental verification.

Recent experimental advancements in simulating the AFM phase transition in the 3D Hubbard model [52] provide a promising new pathway for realizing the exotic Weyl SC state proposed in this study. To facilitate the successful observation of SC, it is essential to effectively suppress the AFM order. This can be achieved through control over carrier doping, interaction strength, or introducing frustration via NNN hopping or structural modifications to the lattice. Critically, these parameters can be controlled and manipulated in optical lattice systems [49–52]. This tunability offers a highly promising experimental avenue for realizing the predicted Weyl TSC state.

## Supplementary Material

nwag326_Supplemental_File
