# Supplementary material for: Octupolar Weyl superconductivity from electron–electron interaction
Source: Natl Sci Rev. 2026 Jun 2;13(15):nwag326. doi: 10.1093/nsr/nwag326 (PMC13455629; doi:10.1093/nsr/nwag326)
Supplement: Supplementary file 1 [file nwag326_supplemental_file.pdf]

Supplemental Material for "Octupolar Weyl Superconductivity from Electron-electron Interaction"

Zhiming Pan,[1,*] Chen Lu,[2,*] Fan Yang,[3,†] and Congjun Wu[4,5,6,7,‡]

[1] Department of Physics, Xiamen University, Xiamen 361005, Fujian, China
[2] School of Physics, Hangzhou Normal University, Hangzhou 311121, China
[3] School of Physics, Beijing Institute of Technology, Beijing 100081, China
[4] New Cornerstone Science Laboratory, Department of Physics, School of Science, Westlake University, Hangzhou 310024, Zhejiang, China
[5] Institute for Theoretical Sciences, Westlake University, Hangzhou 310024, Zhejiang, China
[6] Key Laboratory for Quantum Materials of Zhejiang Province, School of Science, Westlake University, Hangzhou 310024, Zhejiang, China
[7] Institute of Natural Sciences, Westlake Institute for Advanced Study, Hangzhou 310024, Zhejiang, China

CONTENTS

A. Pairing symmetries and Ginzburg-Landau analysis 1

B. Numerical simulation in the weak-coupling regime and the pairing natures 2

C. Strong coupling t-J model and SBMFT 6

D. Variational Monte Carlo simulation 7

E. TRSB $d + id$ Weyl superconductor 9

References 9

A. Pairing symmetries and Ginzburg-Landau analysis

The pairing symmetries on the cubic lattice are classified according to the irreducible representations (IRRPs) of the O_h group in Tab. A1 [1–3], which can be intuitively represented by the lowest order symmetry polynomials. The lower index g corresponds to even parity spin singlet pairing symmetries (e.g. s and d-waves), and u represents odd parity spin triplet pairing symmetries (e.g. p- and f- waves).

(even) Rep.	Basis functions		nodal structure
A_{1g}	1<br>$x^2+y^2+z^2$	s-wave<br>s-wave	full-gap
A_{2g}	$(x^2-y^2)(y^2-z^2)(z^2-x^2)$	i-wave	line-node
E_g	$\{3z^2-r^2, x^2-y^2\}$	d-wave	line- or point-node
T_{1g}	$\{xy(x^2-y^2), \cdots\}$	g-wave	line- or point-node
T_{2g}	$\{xy, yz, zx\}$	d-wave	full-gap or node
(odd) Rep.	Basic $\mathbf{d}$-vector functions		
A_{1u}	$\hat{\mathbf{x}}k_x+\hat{\mathbf{y}}k_y+\hat{\mathbf{z}}k_z$	p-wave	full-gap
A_{2u}	$\hat{\mathbf{x}}k_x(k_z^2-k_y^2)+\cdots$	f-wave	point-node
E_u	$\{2\hat{\mathbf{z}}k_z-\hat{\mathbf{x}}k_x-\hat{\mathbf{y}}k_y, \sqrt{3}(\hat{\mathbf{x}}k_x-\hat{\mathbf{y}}k_y)\}$	p-wave	full-gap or point-node
T_{1u}	$\{\hat{\mathbf{y}}k_z-\hat{\mathbf{z}}k_y, \cdots\}$	p-wave	point-node
T_{2u}	$\{\hat{\mathbf{y}}k_z+\hat{\mathbf{z}}k_y, \cdots\}$	p-wave	full-gap or point-node

TABLE A1. IRRPs of gap functions for the octahedral group O_h, including both the singlet even parity and triplet odd parity channels. The nodal structure for each representation is also shown in the last column. For multi-component representation, the nodal structure could be full gap, nodal point or nodal line depending on the pairing mixing.

For the two-fold degenerate superconductivity (SC) within the E_g IRRP, $(d_{3z^2-r^2}, d_{x^2-y^2})$, the Ginzburg-Landau free energy up to the quartic level takes the following form [3]

$$\mathcal{F} = \alpha\big(|\Delta_1|^2 + |\Delta_2|^2\big) + \beta_1\big(|\Delta_1|^2 + |\Delta_2|^2\big)^2 + \beta_2\big|\Delta_1^*\Delta_2 - \Delta_2^*\Delta_1\big|^2, \tag{A1}$$

where Δ_1, Δ_2 represent the order parameters of $d_{3z^2-r^2}$ and $d_{x^2-y^2}$ pairings, respectively. For $\beta_2 < 0$, the phase difference between the two components is $\pm\frac{\pi}{2}$, leading to a complex $1 : \pm i$ mixing of the pairing functions with equal amplitude. This state exhibits spontaneously time-reversal symmetry breaking (TRSB), resulting in Weyl SC. Conversely, when $\beta_2 > 0$, the two components share the same phase, resulting in an order parameter of the form:

$$\vec{\Delta} = (\Delta_1, \Delta_2) \propto (\cos\theta, \sin\theta) \tag{A2}$$

which corresponds to a nematic state that reduces the full rotational symmetry of the O_h group to its subgroup. This quartic-order Ginzburg-Landau theory exhibits an accidental continuous degeneracy for the nematic state, which can be lifted through the inclusion of higher-order terms. To resolve this degeneracy, a sixth-order term respecting the O_h symmetry in the free energy can be added [3]:

$$\mathcal{F}_\gamma = \gamma_1\big(|\Delta_1|^2 + |\Delta_2|^2\big)^3 + \gamma_2\big(|\Delta_1|^2 + |\Delta_2|^2\big)\big|\Delta_1^2 + \Delta_2^2\big|^2 + \gamma_3|\Delta_1|^2\big|3\Delta_2^2 - \Delta_1^2\big|^2, \tag{A3}$$

where the γ_3 term is proportional to $\cos^2(3\theta)$. It is evident that a positive γ_3 favors the nematic state with $\theta = (2n+1)\pi/6$ $(n \in \mathbb{Z})$, e.g, $(\Delta_1, \Delta_2) \propto (0, 1)$, while a negative γ_3 supports the state with $\theta = 2n\pi/6$ $(n \in \mathbb{Z})$, e.g, $(\Delta_1, \Delta_2) \propto (1, 0)$.

Additionally, the O_h symmetry permits the existence of three-fold degenerate SC state, belonging to the T_{1g}, T_{2g}, T_{1u} or T_{2u} IRRP, as detailed in Tab. A1. They are characterized by three-component order parameters Δ'_a $(a = 1, 2, 3)$. Up to quartic order, the Ginzburg-Landau free energy for these states is expressed as follows [3],

$$\mathcal{F} = \alpha\sum_a |\Delta'_a|^2 + \beta_1\big(\sum_a |\Delta'_a|^2\big)^2 + \beta_2\big|\sum_a \Delta'^2_a\big|^2 + \beta_3\Big(|\Delta'_1|^2|\Delta'_2|^2 + |\Delta'_2|^2|\Delta'_3|^2 + |\Delta'_3|^2|\Delta'_1|^2\Big), \tag{A4}$$

where we require $\alpha < 0$ and $\beta_1 > 0$ in the SC state. The sign of β_2 is crucial in determining the configuration of the SC state: a positive $\beta_2 > 0$ favors a complex mixing structure among the components, where a negative $\beta_2 < 0$ favors a real mixing.

$(\Delta'_1, \Delta'_2, \Delta'_3)$	Free energy	condition	T_{2g} nodal structure	T_{1u} nodal structure
$e^{i\theta}(1, 0, 0)$	$-4\alpha/(\beta_1 + \beta_2)$	$\beta_3 > 0, 4\beta_2 < \beta_3$	line-node	point-node
$e^{i\theta}(1, 1, 0)$	$-4\alpha/(\beta_1 + \beta_2 + \beta_3/4)$	#		
$e^{i\theta}(1, i, 0)$	$-4\alpha/(\beta_1 + \beta_3/4)$	$0 < \beta_3 < 4\beta_2$	line-node	point-node
$e^{i\theta}(1, 1, 1)$	$-4\alpha/(\beta_1 + \beta_2 + \beta_3/3)$	$\beta_2, \beta_3 < 0$	full-gap	point-node
$e^{i\theta}(1, \omega, \omega^2)$	$-4\alpha/(\beta_1 + \beta_3/3)$	$\beta_3 < 0 < \beta_2$	point-node	point-node

TABLE A2. Possible ground state configurations $(\Delta_1, \Delta_2, \Delta_3)$ and their associated free energy. The conditions for each configuration are listed in the third column. The nodal structure corresponding to each state for T_{2g} and T_{1u} IRRR is provided in the final column. The symbol "#" indicates that this state cannot be stabilized as the ground state under any conditions.

The possible ground states and their associated free energies are summarized in Tab. A2. The ground state is attained when the free energy is minimized [3]. Notably, a nematic state featuring only two non-zero components is not favored within the relevant parameter regime. For the case where $\beta_3 < 0 < \beta_2$, a TRSB state emerges as the preferred configuration, with the order parameter taking the complex structure $(\Delta'_1, \Delta'_2, \Delta'_3) \propto (1, e^{\pm i2\pi/3}, e^{\pm i4\pi/3})$.

B. Numerical simulation in the weak-coupling regime and the pairing natures

In the weak-coupling regime, where the perturbative analysis is applicable, the random phase approximation (RPA) is a powerful and well-established theoretical framework employed to analyze SC instabilities. This approach systematically accounts for pairing mediated by collective electronic excitations, specifically investigating charge and spin fluctuations arising from the particle-hole bubble due to the repulsive Hubbard interaction.

The key quantities in the RPA analysis are the dynamic spin and charge susceptibilities. The bare (non-interacting, $U = 0$) susceptibility $\chi_0(\mathbf{q}, i\omega_n)$ is calculated using the single-particle band structure $\varepsilon_\mathbf{k}$:

$$\chi_0(\mathbf{q}, i\omega_n) = \frac{1}{N}\sum_\mathbf{k} \frac{f(\varepsilon_\mathbf{k}) - f(\varepsilon_{\mathbf{k}+\mathbf{q}})}{i\omega_n + \varepsilon_\mathbf{k} - \varepsilon_{\mathbf{k}+\mathbf{q}}}, \qquad \varepsilon_\mathbf{k} = -2t\Big[\cos(k_x) + \cos(k_y) + \cos(k_z)\Big] - \mu,$$

where, N is the number of lattice site, t is the nearest-neighbor hopping amplitude and μ is the chemical potential. $f(\varepsilon)$ denotes the Fermi-Dirac distribution function, and $\omega = (2\pi + 1)n/\beta$ ($\beta = 1/k_B T$, $n \in \mathbb{Z}$) are fermionic Matsubara frequencies. Within RPA, the charge susceptibility $\chi^{(c)}(\mathbf{q}, i\omega_n)$, which describes the system's response to charge density fluctuations, is obtained by summing the geometric series of bubble diagrams connected by the Hubbard interaction U,

$$\chi^{(c)}(\mathbf{q}, i\omega_n) = \frac{\chi_0(\mathbf{q}, i\omega_n)}{1 + U\chi_0(\mathbf{q}, i\omega_n)}, \quad \chi^{(c)}(\mathbf{q}) \equiv \chi^{(c)}(\mathbf{q}, i\omega_n \to 0).$$

Here, we have also introduced static susceptibility $\chi^{(c)}(\mathbf{q})$ in the $i\omega_n \to 0$ limit. The positive sign in the denominator reflects the repulsive nature of the direct Coulomb interaction for charge fluctuations. Similarly, the spin susceptibility $\chi^{(s)}$ is given by,

$$\chi^{(s)}(\mathbf{q}, i\omega_n) = \frac{\chi_0(\mathbf{q}, i\omega_n)}{1 - U\chi_0(\mathbf{q}, i\omega_n)}, \quad \chi^{(s)}(\mathbf{q}) \equiv \chi^{(s)}(\mathbf{q}, i\omega_n \to 0).$$

These collective spin and charge fluctuations mediate an effective pairing interaction $V_{\text{eff}}(\mathbf{k}, \mathbf{q})$ between electrons forming Cooper pairs, as described by the pairing term in the Hamiltonian,

$$H_{\text{pairing}} = \sum_{\mathbf{k},\mathbf{q} \in \text{F.S.S}} V_{\text{eff}}(\mathbf{k}, \mathbf{q}) c^\dagger_{\mathbf{k}\uparrow} c^\dagger_{-\mathbf{k}\downarrow} c_{-\mathbf{q}\downarrow} c_{\mathbf{q}\uparrow}, \tag{A5}$$

where F.S.S denotes a small energy shell windows around the Fermi surface and the main contribution of pairing is dominated from the modes near the Fermi surface. The effective pairing interaction can be decomposed into the singlet $V^{(s)}_{\text{eff}}$ and triplet $V^{(t)}_{\text{eff}}$ channels, which are related to the dynamic charge/spin susceptibilities as follows:

$$\begin{aligned} V^{(s)}_{\text{eff}}(\mathbf{k}, \mathbf{q}) =& U + \frac{3U^2}{4}\Big[\chi^{(s)}(\mathbf{k}+\mathbf{q}) + \chi^{(s)}(\mathbf{k}-\mathbf{q})\Big] - \frac{U^2}{4}\Big[\chi^{(c)}(\mathbf{k}+\mathbf{q}) + \chi^{(c)}(\mathbf{k}-\mathbf{q})\Big], \\ V^{(t)}_{\text{eff}}(\mathbf{k}, \mathbf{q}) =& -\frac{U^2}{2}\Big[\chi^{(s)}(\mathbf{k}+\mathbf{q}) + \chi^{(s)}(\mathbf{k}-\mathbf{q})\Big] - \frac{U^2}{2}\Big[\chi^{(c)}(\mathbf{k}+\mathbf{q}) + \chi^{(c)}(\mathbf{k}-\mathbf{q})\Big] \end{aligned} \tag{A6}$$

From the effective pairing interaction Eq. A5, a self-consistent gap equation can be derived using a mean-field analysis analogous to conventional BCS theory. The superconducting gap function is determined by,

$$\tilde{\Delta}(\mathbf{k}) \equiv \sum_{\mathbf{k},\mathbf{q} \in \text{F.S.S}} V_{\text{eff}}(\mathbf{k}, \mathbf{q}) \langle c_{-\mathbf{q}\downarrow} c_{\mathbf{q}\uparrow} \rangle = -\sum_{\mathbf{q} \in \text{F.S.S}} V_{\text{eff}}(\mathbf{k}, \mathbf{q}) \frac{\tilde{\Delta}(\mathbf{q})}{E_{\mathbf{k}}} \tanh\Big(\frac{\beta}{2} E_{\mathbf{k}}\Big), \tag{A7}$$

where $E_{\mathbf{k}} = \sqrt{\varepsilon^2_{\mathbf{k}} + |\tilde{\Delta}(\mathbf{q})|^2}$ is the Bogoliubov quasiparticle energy. Near the critical temperature T_c, the gap approaches zero, allowing the gap equation to be linearized,

$$\lambda \tilde{\Delta}(\mathbf{k}) = -\frac{1}{(2\pi)^2} \oint_{\text{F.S}} dq_\parallel \frac{V_{\text{eff}}(\mathbf{k}, \mathbf{q})}{v_F(\mathbf{q})} \tilde{\Delta}(\mathbf{q}), \qquad T_c \propto e^{-1/\lambda}.$$

where F.S. denotes the Fermi surface and $q_\parallel$ is the tangent component of $\mathbf{q}$. The dimensionless eigenvalue λ determines the pairing strength. The critical temperature T_c exhibits a BCS-like dependence on the eigenvalue, $T_c \propto e^{-1/\lambda}$. Therefore, the pairing symmetry channel associated with the maximal eigenvalue λ_{max} corresponds to the largest T_c and represents the dominant pairing instability. By numerically solving the RPA equations for the susceptibilities and calculating the corresponding eigenvalues λ for different symmetry channels, one can determine the leading pairing channel.

The associated phase diagram for different doping levels δ based on random-phase approximation (RPA) in the weak-coupling regime is summarized in Fig. A1(a). Near half-filling, a two-fold E_g IRRP within d-wave pairing ($d_{3z^2-r^2}, d_{x^2-y^2}$) is favored. As the doping δ increases, the d-wave pairing symmetry transitions from E_g IRRP to a three-fold degenerate T_{2g} IRRP (d_{xy}, d_{yz}, d_{zx}) at intermediate doping levels. At high doping levels, particularly near $\delta = 1$ with low electron densities, the system exhibits a p-wave triplet pairing corresponding to the T_{1u} IRRP.

To further assess the stability of the E_g pairing near half-filling, we performed comprehensive RPA simulations across a range of interaction strengths U/t. As summarized in Fig. A2, the results clearly demonstrate that the dominant pairing instability near half-filling consistently corresponds to the E_g representation for U/t values ranging

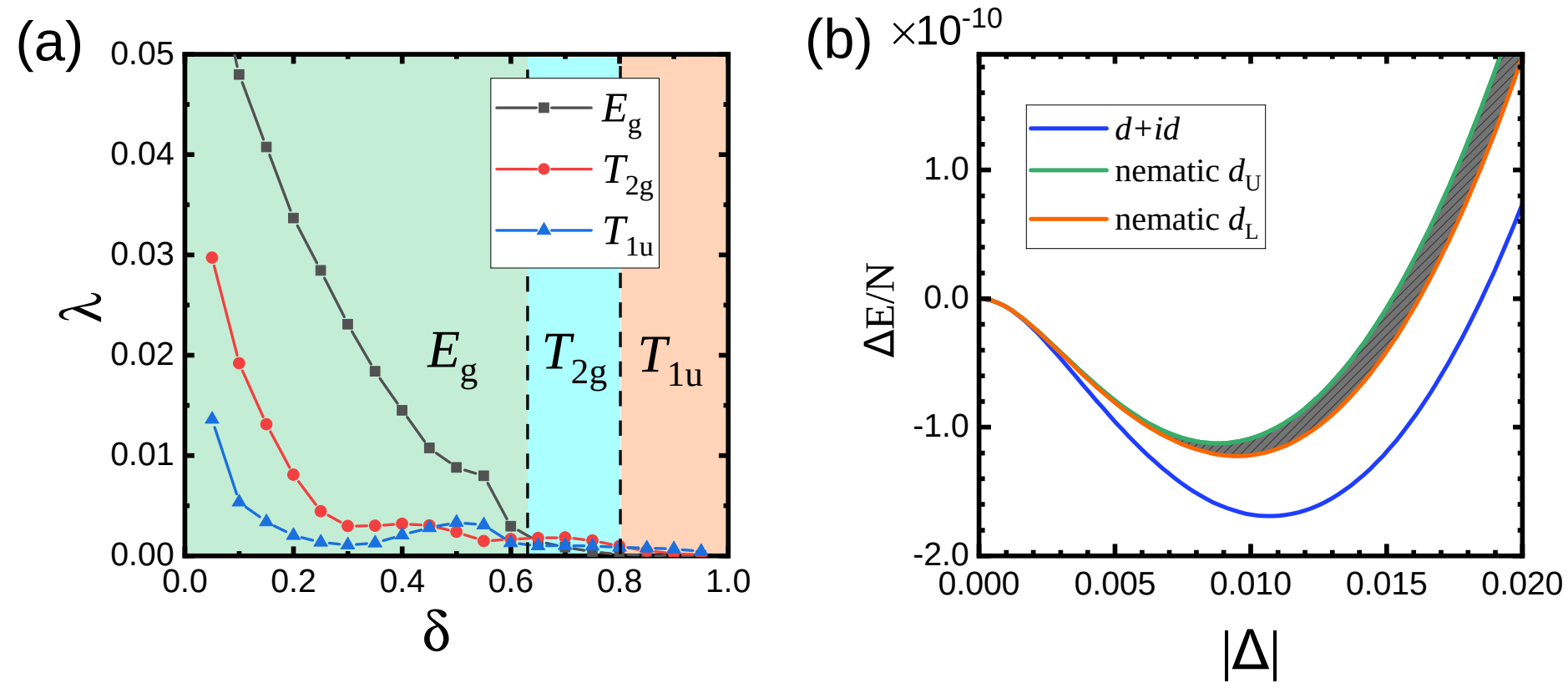

FIG. A1. (a). Dominated pairing strength eigenvalues λ and the phase diagram under doping δ for $U/t = 1.8$. Near half-filling (lower δ), the SC state is dominated by d-wave E_g representation. As the doping level increases, the SC state evolves into the d-wave T_{2g} representation. At higher doping, the SC state is controlled by a p-wave triplet pairing belonging to T_{1u} representation. (b). The mean-field energy gains per site, $\Delta E/N$, relative to the normal state for the TRSB complex $d + id$ pairing and the real nematic d-wave pairings of the E_g representation from RPA study. The parameter values are as follows: doping level $\delta = 0.3$ and $U/t = 3.40$. The horizontal axis, $|\Delta|$, represents the rescaled pairing amplitude, and the physical pairing gap around the Fermi surface is given by $\Delta_{\mathbf{k}} = |\Delta| \cdot f(\mathbf{k})$, where $f(\mathbf{k})$ is the normalized angular form factor for each pairing symmetry. The pairing gap is simulated within an energy shell $\pm 0.02t$ around the Fermi surface, and the pairing amplitude is normalized within this shell. The shaded region corresponds to all possible real nematic pairings with $d_{U,L}$ representing the upper and lower energy bounds of the real nematic pairings. The largest amplitude of the form factor $|f(\mathbf{k})|$ for $d + id$ pairing is given by 1.22×10^{-2}.

from 0.3 to 0.15. This consistency across different interaction strengths strongly corroborates our conclusion from the weak-coupling RPA analysis that E_g d-wave pairing is the favored state near half-filling.

As depicted in Fig. A2, the results also reveal clear trends for the eigenvalue λ associated with the dominant E_g channel: λ generally increases with increasing interaction strength U/t (within the studied range where RPA is applicable). This is physically intuitive, as a stronger bare repulsion U can lead to enhanced collective spin (and charge) fluctuations, which in turn provide a more effective pairing "glue" in appropriate channels. on the contrary, λ decreases with increasing hole doping δ away from half-filling. This behavior likely reflects changes in the Fermi surface geometry and nesting conditions. As doping increases, the system may move away from conditions that optimally favor the specific spin fluctuations (e.g., those peaked at or near AFM wavevectors) that most effectively drive the E_g pairing.

With the spin-fluctuation mediated pairing scenario described by RPA, the emergence of SC is intimately linked to the leading attractive eigenvalue λ. The SC critical temperature T_c is expected to follow an exponential dependence on this eigenvalue, $T_c \propto e^{-1/\lambda}$. The zero-temperature pairing gap amplitude, Δ, which typically scales with T_c (e.g., $\Delta \approx 1.764 k_B T_c$ in BCS theory), will also exhibit similar exponential relation to λ. The pairing gap Δ also exhibits a similar exponential dependence on the eigenvalue, $\Delta \propto e^{-1/\lambda}$. Therefore, the pairing gap Δ is expected to follow the same trend: it strengthens with increasing interaction strength U/t, and weakens with increasing doping δ.

The physical intuition for the emergence of an E_g SC state in the 3D cubic-lattice Hubbard model, strongly indicated by the above RPA analysis, draws a compelling analogy to the well-established $d_{x^2-y^2}$-wave pairing in the 2D square-lattice Hubbard model. Extending this to a 3D cubic system, one might initially consider d-wave pairing functions reflecting symmetries along the Cartesian planes, such as $d_{x^2-y^2}$, $d_{y^2-z^2}$, and $d_{z^2-x^2}$. However, for the classification of pairing states under the cubic (O_h) point group, it is essential to use basis functions that transform according to its IRRPs, as depicted in Tab. A1. The $L = 2$ (d-wave) spherical harmonics decompose under O_h symmetry into a two-fold degenerate E_g IRRP and a three-fold degenerate T_{2g} IRRP. The standard choice for the E_g basis functions, which are central to our findings, are $\{d_{3z^2-r^2}, d_{x^2-y^2}\}$. The $d_{3z^2-r^2}$ function (combined by $d_{y^2-z^2}$ and $d_{z^2-x^2}$) is a specific linear combination that, along with $d_{x^2-y^2}$, correctly spans this two-dimensional E_g space. The T_{2g} representation, for completeness, is spanned by $\{d_{xy}, d_{yz}, d_{zx}\}$. Our RPA calculations, which assess the strength of pairing instabilities mediated by electron-electron correlations, consistently identify the E_g channel as the leading instability at low to intermediate doping levels in this 3D Hubbard model.

We perform the Ginzburg-Landau analysis to the pairing configuration based on this two-component E_g orders (Δ_1, Δ_2), with $\Delta_{1,2}$ representing the $d_{3z^2-r^2}$ and $d_{x^2-y^2}$ pairings, respectively. Up to the quartic order, two types

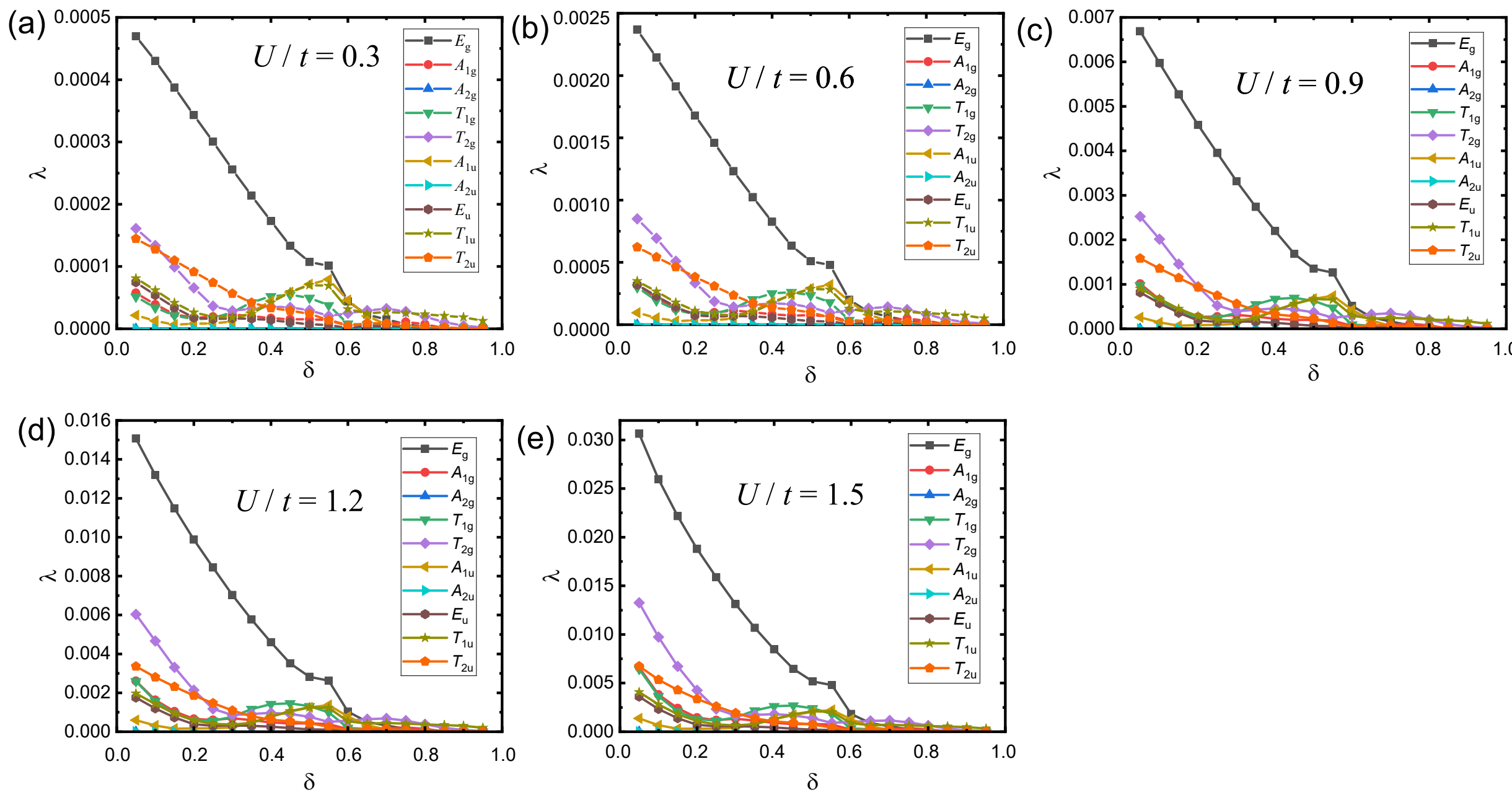

FIG. A2. (a-e) Pairing eigenvalues for different representation from RPA simulations for varying $U/t = 0.3, 0.6, 0.9, 1.2$ and 1.5 as a function of doping δ. The dominant E_g pairing near half-filling (smaller δ) is a consistent feature across these interaction strengths.

of configurations are allowed depending on the sign of the coefficient β_2. The contribution from the β_2 term to the energy is $\beta_2|\Delta_1\Delta_2|^2 \sin^2\Delta\phi$, where $\Delta\phi$ is the phase difference between Δ_1 and Δ_2. For $\beta_2 < 0$, to minimize the energy, it gives rise to $\Delta\phi = \pm\frac{\pi}{2}$ and $|\Delta_1| = |\Delta_2|$, which shows that the $d + id$ type complex mixed pairing is favored. In contrast, for $\beta_2 > 0$, it yields $\Delta\phi = 0$ leaving the ratio between $|\Delta_1|$, $|\Delta_2|$ arbitrary, which corresponds to a real nematic pairing. The degeneracy of the real mixed pairing states will be removed at the 6th order Ginzburg-Landau level analysis (For details, see discussions in S.M. Sec.A).

To further explore these possibilities and identify the energetically favored state, the mean-field calculations subsequent to the RPA study are performed to compare the energies of various pairing states, including the complex $d + id$ pairing with equal amplitude and $\Delta\phi = \pm\frac{\pi}{2}$, and the real one $\cos\theta d_{3z^2-r^2} + \sin\theta d_{x^2-y^2}$ with θ controlling the amplitude ratio. We calculate the configurations for θ ranging from $\theta = 0$ to $\theta = \pi$, which covers all possible real pairings.

Within the mean-field theory, we can study the total energy of the superconducting state by approximating the full pairing Hamiltonian H. The full Hamiltonian consists of the kinetic term and the effective pairing interaction:

$$H = H_t + H_{\text{pairing}} = \sum_{\mathbf{k}\sigma} \varepsilon_{\mathbf{k}} c^\dagger_{\mathbf{k}\sigma} c_{\mathbf{k}\sigma} + \sum_{\mathbf{k},\mathbf{q}\in \text{F.S.S}} V_{\text{eff}}(\mathbf{k},\mathbf{q}) c^\dagger_{\mathbf{k}\uparrow} c^\dagger_{-\mathbf{k}\downarrow} c_{-\mathbf{q}\downarrow} c_{\mathbf{k}\uparrow}.$$

The interaction term is decoupled via the standard MFT procedure, resulting in the mean-field Hamiltonian H_{MF}:

$$H_{\text{MF}} = \sum_{\mathbf{k}\sigma} \varepsilon_{\mathbf{k}} c^\dagger_{\mathbf{k}\sigma} c_{\mathbf{k}\sigma} + \sum_{\mathbf{k}} \left(\Delta_{\mathbf{k}} c^\dagger_{\mathbf{k}\uparrow} c^\dagger_{-\mathbf{k}\downarrow} + \text{H.c.} \right)$$

The ground-state energy E is then approximated by calculating the expectation value of the original Hamiltonian H using the ground state obtained from H_{MF}:

$$E = \langle H \rangle_{\text{MF}} = \sum_{\mathbf{k}\sigma} \varepsilon_{\mathbf{k}} \langle c^\dagger_{\mathbf{k}\sigma} c_{\mathbf{k}\sigma} \rangle_{\text{MF}} + \sum_{\mathbf{k},\mathbf{q}\in \text{F.S.S}} V_{\text{eff}}(\mathbf{k},\mathbf{q}) \langle c^\dagger_{\mathbf{k}\uparrow} c^\dagger_{-\mathbf{k}\downarrow} \rangle_{\text{MF}} \langle c_{-\mathbf{q}\downarrow} c_{\mathbf{q}\uparrow} \rangle_{\text{MF}}$$

under the constraint adjusting the chemical potential implicitly contained within $\varepsilon_{\mathbf{k}}$,

$$\sum_{\mathbf{k}\sigma} \langle c^\dagger_{\mathbf{k}\sigma} c_{\mathbf{k}\sigma} \rangle_{\text{MF}} = 2\left(N_{\varepsilon<\varepsilon_F} - N_{\varepsilon<\varepsilon_F-\delta\varepsilon} \right)$$

In this equation, $\langle\cdots\rangle_{\rm MF}$ denotes the expectation value calculated with respect to the mean-field ground state. The numerical results, presented in Fig. A1(b), illustrate the ground-state energy gain per site as a function of the gap amplitudes for the above-mentioned states, based on the results of the RPA calculation. It shows that the $d+id$ pairing with equal amplitude and $\Delta\phi=\pm\frac{\pi}{2}$, hosts an energy lower than those of all the possible configurations of the real pairings $\cos\theta d_{3z^2-r^2}+\sin\theta d_{x^2-y^2}$, thus is energetically favorable. The energy variations among the real mixed states with varying amplitude ratios, which corresponds to the sixth-order terms in the Ginzburg-Landau free energy, are much smaller than the differences between the complex and real mixed states. This result is in agreement with both the intuitive arguments presented in the main text and the findings in the strong-coupling limit.

At intermediate doping levels, a TRSB superconducting state within the T_{2g} representation may emerge. This state corresponds to the following form of the order parameter:

$$\Delta(\mathbf{k})=\frac{\Delta_0}{\sqrt{3}}\big(k_xk_y+e^{i\frac{2\pi}{3}}k_yk_z+e^{i\frac{4\pi}{3}}k_zk_x\big), \tag{A8}$$

which exhibits an eight-fold degeneracy linked by the mirror symmetry or time-reversal transformation. In this state, the full O_h point group symmetry is reduced to its subgroup. The quasi-particle dispersion for this TRSB T_{2g} state features a total of $6+2$ nodal points, denoted as $\mathbf{k}_{\rm node}$,

$$\mathbf{k}_{\rm node}=k_F(\pm1,0,0),\quad k_F(0,\pm1,0),\quad k_F(0,0,\pm1),\quad \pm k_F(1,1,1).$$

For the TRSB superconducting state in the spin-triplet T_{1u} representation, the d-vector is expressed by:

$$d(\mathbf{k})\propto\hat{\mathbf{x}}(\omega^2k_y-\omega k_z)+\hat{\mathbf{y}}(k_z-\omega^2k_x)+\hat{\mathbf{z}}(\omega k_x-k_y). \tag{A9}$$

with $\omega=e^{i2\pi/3}$. This TRSB state is non-unitary, exhibiting net spin polarization due to finite $\mathbf{q}=i\mathbf{d}\times\mathbf{d}^*$. Moreover, it also exhibits nodal points.

Additionally, the three-fold degenerate SC represented by the three components pairing gaps $(\Delta_1',\Delta_2',\Delta_3')$ may realize a sextetting order, where six electrons combine to form a bound state, commonly referred to as charge-$6e$ order. Intriguingly, the p-wave triplet representation T_{1u} might realize a sextetting pairing with distinct angular momentum characteristics, differing from the singlet sextetting pairing within the T_{2g} representation.

C. Strong coupling t-J model and SBMFT

In the strong coupling limit, the low-energy effective theory is described by a t-J model under the no-double occupancy constraint,

$$H_{tJ}=-t\sum_{\langle i,j\rangle\sigma}\mathcal{P}\big(c_{i\sigma}^\dagger c_{j\sigma}+\text{h.c.}\big)\mathcal{P}+J\sum_{\langle i,j\rangle}\Big(\mathbf{S}_i\cdot\mathbf{S}_j-\frac{1}{4}n_in_j\Big), \tag{A10}$$

where $\mathbf{S}_i=\frac{1}{2}c_i^\dagger\sigma c_i$ is the spin operator at lattice site i and $J=4t^2/U$ is the nearest-neighbor (NN) antiferromagnetic (AFM) spin superexchange in the large-U limit. The operator $\mathcal{P}$ projects out double-occupied states, reflecting strong correlation effects.

The constrained t-J model can be analyzed using slave-boson mean-field theory (SBMFT) [4, 5]. In this framework, the bare electron operators are expressed in terms of auxiliary holon and spinon operators, maintaining the no-double occupancy constraint:

$$c_{i\sigma}^\dagger\to f_{i\sigma}^\dagger h_i,\qquad c_{i\sigma}\to h_i^\dagger f_{i\sigma},\qquad \sum_\sigma f_{i\sigma}^\dagger f_{i\sigma}+h_i^\dagger h_i=1, \tag{A11}$$

where $h_i^\dagger$ and $f_{i\sigma}^\dagger$ are the holon and spinon creation operators at the lattice site i, respectively. In the ground state, the holon condenses, allowing us to replace the holon operator with its condensation density $h_i=h_j^\dagger=\sqrt{\delta}$.

Within the mean-field theory, the following spinon hopping and pairing order parameters are introduced,

$$\chi_{\mathbf{ij}}=\langle f_{\mathbf{j}\uparrow}^\dagger f_{\mathbf{i}\uparrow}+f_{\mathbf{j}\downarrow}^\dagger f_{\mathbf{i}\downarrow}\rangle\equiv\chi_{\mathbf{j}-\mathbf{i}},\qquad \tilde{\Delta}_{\mathbf{ij}}=\langle f_{\mathbf{j}\downarrow}f_{\mathbf{i}\uparrow}-f_{\mathbf{j}\uparrow}f_{\mathbf{i}\downarrow}\rangle\equiv\tilde{\Delta}_{\mathbf{j}-\mathbf{i}}, \tag{A12}$$

assuming translational symmetry, such that the order parameters depend only on the relative distance between sites. We focus on the NN hoppings and pairings along the three directions $\mu=x,y,z$, i.e., $\chi_\mu=\chi_{\mathbf{i},\mathbf{i}+\hat{\mu}}$ and $\tilde{\Delta}_\mu=\tilde{\Delta}_{\mathbf{i},\mathbf{i}+\hat{\mu}}$. The spin-exchange can be decomposed as follows:

$$J\mathbf{S}_i\cdot\mathbf{S}_j=-\frac{3}{8}J\Big[\chi_{ij}\big(f_{i\uparrow}^\dagger f_{j\uparrow}+f_{i\downarrow}^\dagger f_{j\downarrow}\big)+\text{h.c.}\Big]-\frac{3}{8}J\Big[\tilde{\Delta}_{ij}\big(f_{i\uparrow}^\dagger f_{j\downarrow}^\dagger-f_{i\downarrow}^\dagger f_{j\uparrow}^\dagger\big)+\text{h.c.}\Big]+\text{const.} \tag{A13}$$

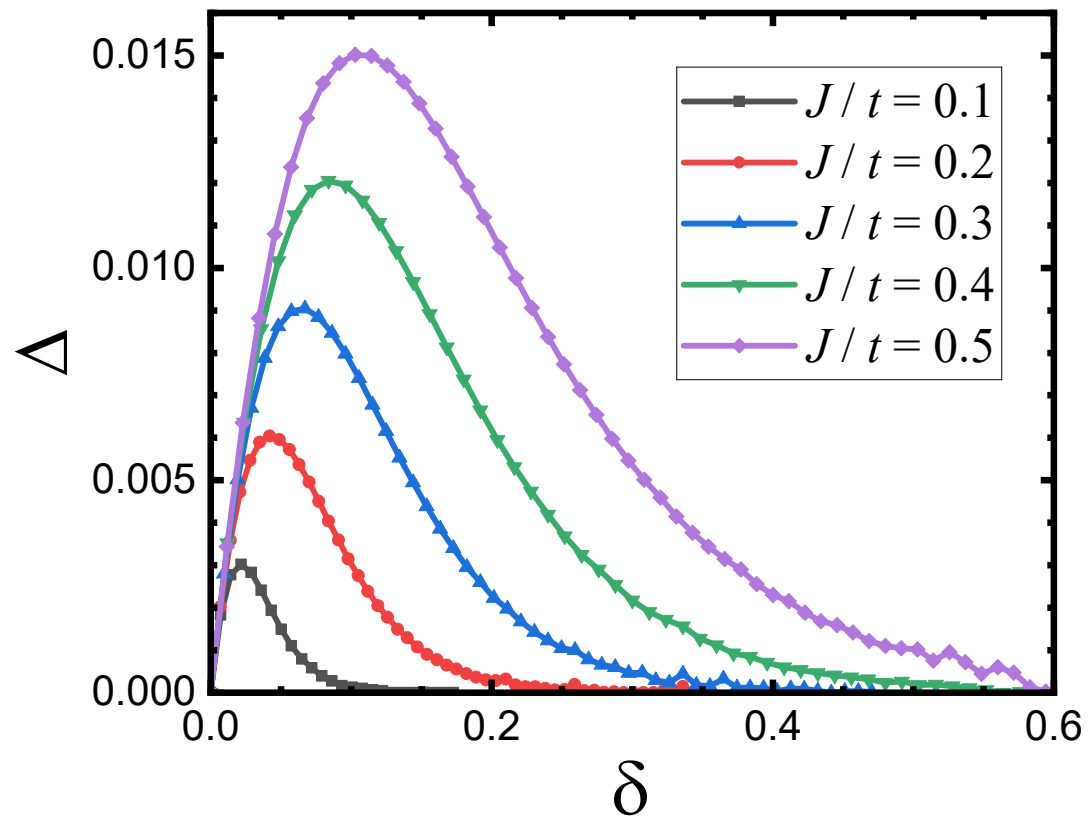

FIG. A3. Pairing amplitude for the t-J model based on the slave-boson mean-field calculation, for a range of $J/t = 0.1, 0.2, 0.3, 0.4, 0.5$

The slave-boson mean-field Hamiltonian for the spinon field in momentum space is expressed as:

$$H_{f,\mathrm{MF}} = \sum_{\mathbf{k}\sigma} \varepsilon(\mathbf{k}) f^\dagger_{\mathbf{k}\sigma} f_{\mathbf{k}\sigma} + \sum_{\mathbf{k}} \Big[\tilde{\Delta}(\mathbf{k}) \big(f^\dagger_{\mathbf{k}\uparrow} f^\dagger_{-\mathbf{k}\downarrow} - f^\dagger_{\mathbf{k}\downarrow} f^\dagger_{-\mathbf{k}\uparrow} \big) + \mathrm{h.c.} \Big] \tag{A14}$$

where the kinetic energy and spinon pairing field are given by

$$\varepsilon(\mathbf{k}) = -2\Big(t\delta + \frac{3}{8}J\chi\Big)\sum_{\mu}\cos(k_\mu) - \mu, \qquad \tilde{\Delta}(\mathbf{k}) = -\frac{3}{8}J\sum_{\mu}\tilde{\Delta}_\mu \cos(k_\mu).$$

Here, we assume a symmetric hopping fields with $\chi_x = \chi_y = \chi_z \equiv \chi$. The physical superconducting order parameter is represented as $\Delta \propto \delta\tilde{\Delta}$, where δ is the holon condensation density.

The pairing configuration $(\Delta_x, \Delta_y, \Delta_z) \propto (1, 1, 1)$ corresponds to a s-wave pairing. In contrast, the pairing configuration $(\Delta_x, \Delta_y, \Delta_z) \propto (1, -1, 0)$ and $(\Delta_x, \Delta_y, \Delta_z) \propto (-1, -1, 2)$ correspond to the $d_{x^2-y^2}$-wave pairing and $d_{3z^2-r^2}$, respectively, both expected to be degenerate under the E_g representation of the O_h symmetry group.

The self-consistent SBMFT gap equations for the nearest-neighbor spinon pairing amplitudes $\tilde{\Delta}_\mu$ ($\mu = x, y, z$) are then solved:

$$\tilde{\Delta}_\mu = \langle f_{\mathbf{i}+\hat{\mu}\downarrow} f_{\mathbf{i}\uparrow} - f_{\mathbf{i}+\hat{\mu}\uparrow} f_{\mathbf{i}\downarrow} \rangle_{\mathrm{MF}} = \frac{2}{N}\sum_{\mathbf{k}} \cos(k_\mu) \langle f_{\mathbf{k}\downarrow} f_{-\mathbf{k}\uparrow} \rangle_{\mathrm{MF}}$$

where the expectation value $\langle \cdots \rangle_{\mathrm{MF}}$ is calculated with respect to the spinon mean-field Hamiltonian $H_{f,\mathrm{MF}}$. Within a relevant range of parameter regime (doping levels δ and exchange coupling J/t), the numerical results indicate that a d-wave superconducting state, characterized by a complex phase structure $(\Delta_x, \Delta_y, \Delta_z) = \delta\tilde{\Delta}_0(1, e^{\pm i2\pi/3}, e^{\pm i4\pi/3})$, is energetically favored. The resulting overall physical gap amplitude $\Delta_0 = \delta\tilde{\Delta}_0$ (where δ is the hole doping concentration representing the holon condensate density) obtained from these simulations is summarized as a function of doping for various J/t values in Fig. A3.

D. Variational Monte Carlo simulation

To further validate the findings from SBMFT in the strong-coupling regime, where the Hubbard model maps to the t-J model, we employ Variational Monte Carlo (VMC) simulations.

The trial wavefunction for a pairing state is constructed using a Gutzwiller-projected BCS function:

$$|G\rangle = \hat{P}_G\Big(\sum_{\mathbf{k}} \frac{v_{\mathbf{k}}}{u_{\mathbf{k}}} c^\dagger_{\mathbf{k}\uparrow} c^\dagger_{-\mathbf{k}\downarrow}\Big)^{N_e/2} |\mathrm{vac}\rangle, \qquad \frac{v_{\mathbf{k}}}{u_{\mathbf{k}}} = \frac{\Delta(\mathbf{k})}{\varepsilon(\mathbf{k}) + \sqrt{\varepsilon(\mathbf{k})^2 + \Delta(\mathbf{k})^2}}$$

where $\hat{P}_G$ is the Gutzwiller projection operator that enforces the no-double-occupancy constraint at each site and fixes the total particle number N_e for a given hole doping level δ. Consequently, the resulting physical Hilbert space

Pairings	Form factor $f(\mathbf{k})$
s	$2\cos(k_z)+2\cos(k_x)+2\cos(k_y)$
$d_{3z^2-r^2}+id_{x^2-y^2}$	$2\cos(k_z)+2e^{i2\pi/3}\cos(k_x)+2e^{i4\pi/3}\cos(k_y)$
$d_{3z^2-r^2}$	$2\cos(k_z)-\cos(k_x)-\cos(k_y)$
$d_{x^2-y^2}$	$2\cos(k_x)-2\cos(k_y)$
d_{xy}, d_{yz}, d_{zx}	$2\cos(k_x+k_y)+2\cos(k_x-k_y)$
p_x, p_y, p_z	$2\sin(k_x)$

TABLE A3. Gap functions for the VMC calculations

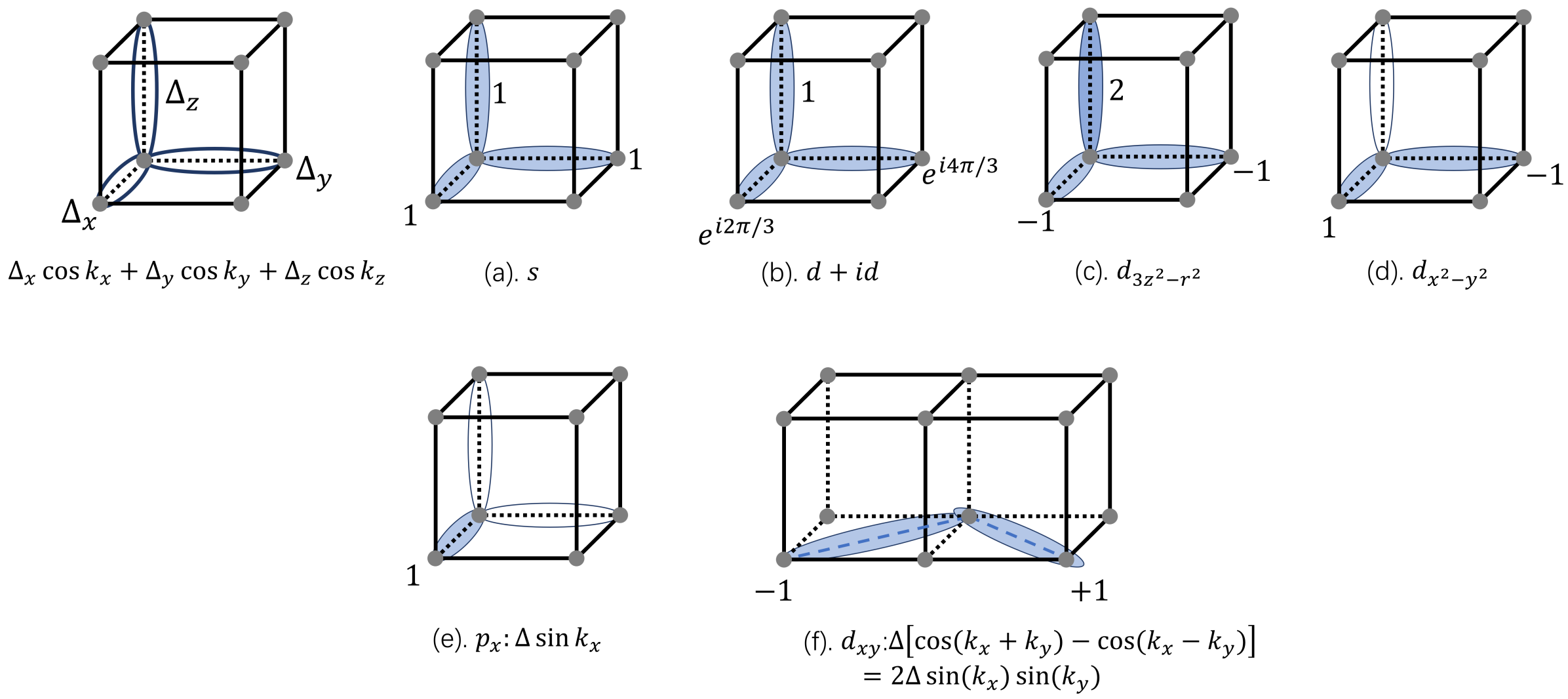

FIG. A4. Real-space pairing configurations for selected superconducting states. Panels (a-d) display different nearest-neighbor pairing symmetries, described by the form $\Delta_x\cos(k_x)+\Delta_y\cos(k_y)+\Delta_z\cos(k_z)$. The numbers on the bonds represent the relative complex amplitudes (magnitude and phase) of the nearest-neighbor pairing parameters. Panel (e) illustrates p_x pairing, and (f) shows next-nearest-neighbor d_{xy} pairing.

allows only three local configurations: empty (hole), singly occupied with spin up, or singly occupied with spin down. The coefficients $u_{\mathbf{k}}$ and $v_{\mathbf{k}}$, defining the underlying BCS function, are related to the dispersion $\varepsilon(\mathbf{k})$ and the pairing gap $\Delta(\mathbf{k})$. $\varepsilon(\mathbf{k})$ represents the kinetic energy dispersion of the non-interacting electrons:

$$\varepsilon_{\mathbf{k}} = -2t\Big[\cos(k_x)+\cos(k_y)+\cos(k_z)\Big]-\mu,$$

The pairing gap function $\Delta(\mathbf{k})=\Delta f(\mathbf{k})$ in the trial wavefunction is chosen to correspond to specific IRRPs of the O_h group, as shown in Tab. A3. Here, the form factor $f(\mathbf{k})$ incorporates either nearest-neighbor or next-nearest-neighbor pairing configurations, whose spatial arrangements for various superconducting states are visually depicted in Fig. A4.

The VMC method utilizes the variational principle to estimate the ground-state energy E. For a fixed pairing amplitude Δ, the optimal ground-state energy of the system for a given pairing configuration is calculated as the expectation value of the t-J Hamiltonian: $E=\langle G|H_{tJ}|G\rangle/\langle G|G\rangle$. In this process, the chemical potential μ is treated as a variational parameter and is systematically adjusted to minimize the total energy E. The comparative energy results for different pairing symmetries obtained from VMC are presented in the main text (Fig. 1(c)). Noticing that antiferromagnetic superexchange J-term acts only between nearest neighbors, naturally favoring singlet and more local pairing mechanisms, we incorporate nearest-neighbor and next-nearest-neighbor range pairing configurations, which are physically relevant. Other possibilities listed in Tab. A1 were also tested but are not depicted, as they resulted in higher energies than the normal state or the d states.

E. TRSB $d+id$ Weyl superconductor

The TRSB Weyl SC pairing characterized by $d_{3z^2-r^2} \pm i d_{x^2-y^2}$ is expressed as follows:

$$\Delta_\eta(\mathbf{k}) = \Delta_0\Big(\cos(k_x) + e^{i\eta\frac{2\pi}{3}} \cos(k_y) + e^{i\eta\frac{4\pi}{3}} \cos(k_z)\Big)$$
$$=\Delta_0\Big(\cos(k_x) - \frac{1}{2}\cos(k_y) - \frac{1}{2}\cos(k_z)\Big) + \Delta_0 i\eta\frac{\sqrt{3}}{2}\Big(\cos(k_y) - \cos(k_z)\Big),$$

where $\eta = \pm 1$ corresponds to the two choices of chirality. This pairing belongs to the E_g irreducible representation of the O_h point group. The Bogoliubov-de Gennes (BdG) Hamiltonian can be formulated in the two-component Nambu spinor representation as follows:

$$H_{\rm BdG} = \sum_{\mathbf{k}} \begin{pmatrix} c^\dagger_{\mathbf{k}\uparrow} & c_{-\mathbf{k}\downarrow} \end{pmatrix} H(\mathbf{k}) \begin{pmatrix} c_{\mathbf{k}\uparrow} \\ c^\dagger_{-\mathbf{k}\downarrow} \end{pmatrix}, \qquad H(\mathbf{k}) = \begin{pmatrix} \varepsilon(\mathbf{k}) & \Delta(\mathbf{k}) \\ \Delta^*(\mathbf{k}) & -\varepsilon(\mathbf{k}) \end{pmatrix}. \tag{A15}$$

Here, the kinetic energy is given by

$$\varepsilon(\mathbf{k}) = -t\Big(\cos(k_x) + \cos(k_y) + \cos(k_z)\Big) - \mu$$

where t represents the effective hopping integral. The Hamiltonian matrix can be expressed in a compact form,

$$H(\mathbf{k}) = \varepsilon(\mathbf{k})\tau_3 + \mathrm{Re}\Delta(\mathbf{k})\tau_1 - \mathrm{Im}\Delta(\mathbf{k})\tau_2 \tag{A16}$$

with τ_i $(i = 1, 2, 3)$ denoting the Pauli matrices in the Nambu particle-hole space.

The Fermi surface is defined by the condition $\varepsilon(\mathbf{k}_F) = 0$, with the eight node points corresponding to:

$$\mathbf{k}_{\rm node} = \frac{1}{\sqrt{3}} k_F(\pm1, \pm1, \pm1),$$

To investigate these nodes further, we focus on the low-energy effective theory around the two points $\mathbf{k}_{111} = (1,1,1)k_F/\sqrt{3}$ and $\mathbf{k}_{\bar{1}\bar{1}\bar{1}} = -(1,1,1)k_F/\sqrt{3}$,

$$H_\eta(\mathbf{k}_{111} + \mathbf{p}) \approx \Big(\varepsilon(\mathbf{k}_{111} + \mathbf{p}) - \varepsilon(\mathbf{k}_{111})\Big)\tau_3 + \Delta_0 \sin\frac{k_F}{\sqrt{3}}\Big(- p_x + \frac{1}{2}p_y + \frac{1}{2}p_z\Big)\tau_1 + \eta\Delta_0\frac{\sqrt{3}}{2}\big(- p_y + p_z\big)\tau_2,$$
$$H_\eta(\mathbf{k}_{\bar{1}\bar{1}\bar{1}} + \mathbf{p}) \approx \Big(\varepsilon(\mathbf{k}_{\bar{1}\bar{1}\bar{1}} + \mathbf{p}) - \varepsilon(\mathbf{k}_{\bar{1}\bar{1}\bar{1}})\Big)\tau_3 - \Delta_0 \sin\frac{k_F}{\sqrt{3}}\Big(- p_x + \frac{1}{2}p_y + \frac{1}{2}p_z\Big)\tau_1 - \eta\Delta_0\frac{\sqrt{3}}{2}\big(- p_y + p_z\big)\tau_2$$

where the kinetic part near the Fermi surface expands to:

$$\varepsilon(\mathbf{k}_{111} + \mathbf{p}) - \varepsilon(\mathbf{k}_{111}) \approx \tilde{t}\sin\frac{k_F}{\sqrt{3}}(p_x + p_y + p_z), \qquad \varepsilon(\mathbf{k}_{\bar{1}\bar{1}\bar{1}} + \mathbf{p}) - \varepsilon(\mathbf{k}_{\bar{1}\bar{1}\bar{1}}) \approx -\tilde{t}\sin\frac{k_F}{\sqrt{3}}(p_x + p_y + p_z)$$

Using an appropriate rescaling of the coordinates, we can write the effective low-energy Hamiltonian as a Weyl form:

$$\begin{aligned} H_\eta(\mathbf{k}_{111} + \mathbf{p}) &\approx + \Big[(\mathbf{v}_1 \cdot \mathbf{p})\tau_1 + \eta(\mathbf{v}_2 \cdot \mathbf{p})\tau_2 + (\mathbf{v}_3 \cdot \mathbf{p})\tau_3\Big], \\ H_\eta(\mathbf{k}_{\bar{1}\bar{1}\bar{1}} + \mathbf{p}) &\approx - \Big[(\mathbf{v}_1 \cdot \mathbf{p})\tau_1 + \eta(\mathbf{v}_2 \cdot \mathbf{p})\tau_2 + (\mathbf{v}_3 \cdot \mathbf{p})\tau_3\Big] \end{aligned} \tag{A17}$$

where the three unit directional vectors are defined as:

$$\mathbf{v}_1 = v\sqrt{\frac{2}{3}}\Big(-1, \frac{1}{2}, \frac{1}{2}\Big), \quad \mathbf{v}_2 = v\eta\frac{1}{\sqrt{2}}\Big(0, -1, 1\Big) \quad \mathbf{v}_3 = v\frac{1}{\sqrt{3}}\Big(1, 1, 1\Big)$$

and $v > 0$ is the effective fermi velocity. The magnetic monopole charge in the momentum space associated with these two Weyl points can be calculated as follows:

$$C_\eta(\mathbf{k}_{111}) = \mathrm{Sgn}\Big[\mathbf{v}_1 \cdot \big(\mathbf{v}_2 \times \mathbf{v}_3\big)\Big] = \eta, \quad C_\eta(\mathbf{k}_{\bar{1}\bar{1}\bar{1}}) = \mathrm{Sgn}\Big[\mathbf{v}_1 \cdot \big(\mathbf{v}_2 \times \mathbf{v}_3\big)\Big] = -\eta.$$

---

* These two authors contributed equally to this work.

† yangfan_blg@bit.edu.cn
‡ wucongjun@westlake.edu.cn

[1] G. Volovik and L. Gor'kov, An unusual superconductivity in ube13, JETP Lett. **39**, 550 (1984).
[2] G. Volovik and L. P. Gor'kov, Superconducting classes in heavy-fermion systems, JETP **61**, 843 (1985).
[3] M. Sigrist and K. Ueda, Phenomenological theory of unconventional superconductivity, Rev. Mod. Phys. **63**, 239 (1991).
[4] G. Kotliar and J. Liu, Superexchange mechanism and *d*-wave superconductivity, Phys. Rev. B **38**, 5142 (1988).
[5] P. A. Lee, N. Nagaosa, and X.-G. Wen, Doping a mott insulator: Physics of high-temperature superconductivity, Rev. Mod. Phys. **78**, 17 (2006).
